# TransCell: *In Silico* Characterization of Genomic Landscape and Cellular Responses by Deep Transfer Learning

**DOI:** 10.1093/gpbjnl/qzad008

**Published:** 2024-09-06

**Authors:** Shan-Ju Yeh, Shreya Paithankar, Ruoqiao Chen, Jing Xing, Mengying Sun, Ke Liu, Jiayu Zhou, Bin Chen

**Affiliations:** Department of Pediatrics and Human Development, Michigan State University, Grand Rapids, MI 49503, USA; Department of Pediatrics and Human Development, Michigan State University, Grand Rapids, MI 49503, USA; Department of Pharmacology and Toxicology, Michigan State University, Grand Rapids, MI 49503, USA; Department of Pediatrics and Human Development, Michigan State University, Grand Rapids, MI 49503, USA; Department of Computer Science and Engineering, Michigan State University, East Lansing, MI 48824, USA; Department of Pediatrics and Human Development, Michigan State University, Grand Rapids, MI 49503, USA; Department of Computer Science and Engineering, Michigan State University, East Lansing, MI 48824, USA; Department of Pediatrics and Human Development, Michigan State University, Grand Rapids, MI 49503, USA; Department of Pharmacology and Toxicology, Michigan State University, Grand Rapids, MI 49503, USA; Department of Computer Science and Engineering, Michigan State University, East Lansing, MI 48824, USA

**Keywords:** Genomics, Transcriptomics, Cancer dependency, Drug repurposing, Transfer learning

## Abstract

Gene expression profiling of new or modified cell lines becomes routine today; however, obtaining comprehensive molecular characterization and cellular responses for a variety of cell lines, including those derived from underrepresented groups, is not trivial when resources are minimal. Using gene expression to predict other measurements has been actively explored; however, systematic investigation of its predictive power in various measurements has not been well studied. Here, we evaluated commonly used machine learning methods and presented TransCell, a two-step deep transfer learning framework that utilized the knowledge derived from pan-cancer tumor samples to predict molecular features and responses. Among these models, TransCell had the best performance in predicting metabolite, gene effect score (or genetic dependency), and drug sensitivity, and had comparable performance in predicting mutation, copy number variation, and protein expression. Notably, TransCell improved the performance by over 50% in drug sensitivity prediction and achieved a correlation of 0.7 in gene effect score prediction. Furthermore, predicted drug sensitivities revealed potential repurposing candidates for new 100 pediatric cancer cell lines, and predicted gene effect scores reflected *BRAF* resistance in melanoma cell lines. Together, we investigated the predictive power of gene expression in six molecular measurement types and developed a web portal (http://apps.octad.org/transcell/) that enables the prediction of 352,000 genomic and cellular response features solely from gene expression profiles.

## Introduction

**Figure 1:**
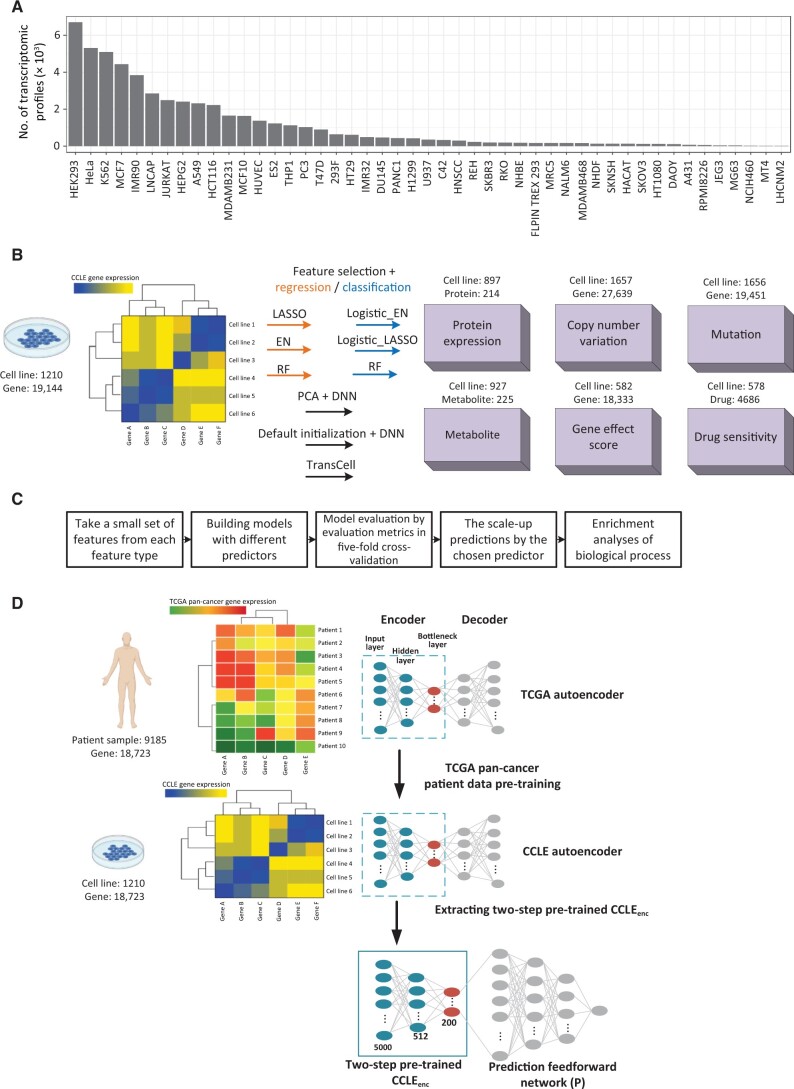
An overview of research design **A**. The number of RNA-seq profiles available per cell line. The numbers were collected from the ARCHS4 website (https://maayanlab.cloud/archs4/). **B**. Prediction of measurements in six types based on gene expression data of cancer cell lines. The number of cell lines varies across data types. **C**. Model evaluation process. Due to the high demand for computation power, we started with a small set of measurements for each type and then scaled up to a larger set. **D**. Schematic of TransCell. The top 5000 features sharing similar distribution between CCLE and TCGA were first selected, followed by the creation of an autoencoder using TCGA pan-cancer tumor transcriptomes. The parameters of the TCGA encoder were then transferred to the second CCLE autoencoder for weight initializations. Afterward, a two-step pre-trained CCLE_enc_ was extracted and linked to a prediction feedforward network. Parameters were tuned automatically (see Method for details). Note that one model is built for each molecular measurement. LASSO, least absolute shrinkage and selection operator; EN, elastic net; RF, random forest; PCA, principal component analysis; DNN, deep neural network; CCLE, Cancer Cell Line Encyclopedia; TCGA, The Cancer Genome Atlas; CCLE_enc_, CCLE encoder.

*In vitro* cell lines are widely used in biomedical research and drug discovery [1–3]. New cell lines are emerging rapidly thanks to the advances in the cell culture of individual tumors. In addition, existing cell lines are frequently modified, such as drug treatment in resistance mechanism elucidation and gene knock-out in therapeutic target identification, leading to many variations of cell lines that may present different molecular profiles from the parental cell lines. Small modification of a cell line may result in a dramatic change of its genomic landscape or even the outcome of its responses to perturbagens. A survey of the RNA sequencing (RNA-seq) repository ARCHS4 [4] indicated that some cell lines even have thousands of gene expression profiles (**Figure 1**A). Obtaining comprehensive molecular measurements of these cell lines would greatly assist research; however, the high cost of experiments does not allow to do so most of the time. Moreover, historically molecular measurements of the cell lines derived from underrepresented groups (*e.g.*, age: pediatric; race: African American and American Indian) are scarce.

The recent efforts in large-scale profiling of common cancer cell lines provide an outstanding reference for cancer research. Outstanding examples include DepMap, a collection of projects such as Cancer Cell Line Encyclopedia (CCLE), PRISM, and Achilles, where transcriptomic, proteomic, metabolomic, and genomic features of over 1000 cancer cell lines, as well as genetic screening and drug screening in these cell lines, were recently profiled [5]. However, such resources are not applicable to the understudied or modified cell lines. RNA, as an intermediate product between DNA and protein, is known to encode information of other measurements (even including potential responses to external agents). If a computational model could decode the information based solely on gene expression, then *in silico* characterization of genomic landscape and cellular responses from gene expression for diverse cell lines is within reach.

Using gene expression to predict the individual measurement types has been actively explored. To achieve optimal performance, genomics and other omics data are frequently borrowed in the prediction of drug sensitivity [6–10], drug synergy [11–13], and gene essentiality [14–16]. Gene expression, mutation, and copy number variation (CNV) were also combined in drug response prediction [17]. Moreover, in drug-related tasks, irregular or non-Euclidean drug structures could be embedded through convolution neural networks (CNNs) [9,18,19] and graph representations [20–22] to aid the modeling. Nevertheless, gene expression consistently shows the most informative in drug response prediction [23,24], whereas mutation and CNV profiles contribute little to improve the accuracy [25]. Although the integration of multiple omics profiles could improve the learning performance, its application is limited in practice where only gene expression data is often accessible. Nevertheless, the aforementioned studies inspired us to perform a systematic investigation of the power of using gene expression in the prediction of various measurement types available in the recent DepMap collection.

In this study, using DepMap data, we comprehensively evaluated commonly used feature selection and baseline machine learning methods in the prediction of protein expression, CNV, mutation, metabolite, gene effect score (a measurement of cancer genetic dependency from CRISPR screening), and drug sensitivity (Figure 1B and C). To address the big p (features) and little n (samples) problem, we further developed TransCell, a deep transfer framework that leverages a pre-training model from large pan-cancer patient profiles to gain better weight initializations. Comparing to train deep neural network (DNN) directly, we demonstrated the superiority of TransCell, which could overcome premature convergence and overfitting under a relatively small sample size. Notably, many outstanding efforts have been made toward using DepMap to predict individual measurement types, *e.g.*, drug sensitivity [10,26], metabolite [27], and gene effect [28]. To improve performance in individual tasks, data other than gene expression were often required in these models, thus it is often challenging to apply them to predict different measurement types. Here, we did not intend to perform head to head comparisons with the published methods across all the measurement types, though we compared a few representative models in the discussion. Moreover, we observed the remarkable performance of the current model and demonstrated its usefulness through a few case studies. We further developed the TransCell web portal (http://apps.octad.org/transcell/), enabling researchers to predict six molecular measurement types based solely on gene expression.

## Method

### Dataset

The cancer cell line log_2_-transformed transcript per million (TPM) gene expression matrix for protein-coding genes (processed by RSEM [29]), including 1210 cell lines and 19,144 genes was downloaded from the DepMap portal (http://www.depmap.org) [5]. The Cancer Genome Atlas (TCGA) pan-cancer RSEM-processed gene expression matrix, including 9185 patient samples and 18,723 genes, was downloaded from Open Cancer Therapeutic Discovery (OCTAD; http://octad.org) [30]. The measurements, including protein expression, CNV, metabolite, gene effect score, drug sensitivity, and mutation, were downloaded from the DepMap portal (Figure 1B) [31–33]. The DepMap 20Q1 serving as a prospective dataset was downloaded from this link: https://figshare.com/articles/dataset/DepMap_20Q1_Public/11791698. For new RNA-seq profiles, we downloaded the raw sequences from the Sequence Read Archive (SRA) and used the OCTAD pipeline to compute TPM [30].

### Transfer learning between TCGA and CCLE

TransCell is composed of two networks: (1) a two-step pre-trained CCLE encoder (CCLE_enc_) and (2) a prediction feedforward network (P) (Figure 1D). The first component is an encoder extracted from the second autoencoder trained by CCLE, transforming higher-order features of CCLE gene expression into a lower-dimensional representation. The encoded representation is then linked to P, resulting in a fully connected network for training. We adopted a parameter-based transfer learning, where the weights learned from the source domain are transferred to the target domain for weight initializations, followed by finetuning of all layers. One major concern of transfer learning is negative transfer, meaning that the source domain needs to resemble the target domain; otherwise, the attempt to transfer knowledge from the source can have a negative impact on the target learner and thus can even lower the performance [34]. Considering feature distribution within source domain (*e.g.*, TCGA) and target domain (*e.g.*, CCLE) for minimizing the effects of negative transfer, we performed a two-sample Kolmogorov–Smirnov test for each gene feature to identify genes with a similar or identical distribution between TCGA and CCLE. According to adjusted *P* values, we chose the top 5000 genes to be our features for training TransCell.

### Two-step pre-training of CCLE_enc_

Autoencoder, a nonlinear unsupervised learning, comprises a symmetric pair of an encoder and a decoder. Given a dataset *X* ($X=\{x_{1},\ldots ,x_{n}\}$) with *n* samples and *m* features, the encoder is a function *f* that maps the input *X* to a lower-dimensional representation, which could be described as below:

(1) $$h=f(X)=s_{f}(WX\;+\;b_{X})$$

where *h* represents the hidden representation of *X*, and $s_{f}$ stands for LeakyReLU, a nonlinear activation function making autoencoder perform a nonlinear projection [35]. The encoder is parameterized by a weight matrix *W* and a bias vector $b_{X}$. Moreover, the decoder function *g* maps the hidden representation *h* back to a reconstruction $\hat{X}$. The process is formulated in the following:

(2) $$\hat{X}=g(h)=s_{g}(W\prime h\;+\;b_{h})$$

where $s_{g}$ is an identity. The decoder’s parameters comprise a weight matrix W′ and a bias vector $b_{h}$. The training process of an autoencoder aims to find a parameter set that minimizes the reconstruction loss on the given data dataset *X*. The objective function is given as:

(3) $$\Phi =\min_{\theta }L(X,\hat{X})=\frac{1}{n}\sum_{i=1}^{n}{\left\|x_{i}\;-\;{\hat{x}}_{i}\right\|}^{2}$$

By minimizing the loss between the original input *X* and reconstruction $\hat{X}$ in Equation 3, an autoencoder reduces the dimension of complex input features and produces a compressed representation at the bottleneck layer, the layer between encoder and decoder. We pre-trained an encoder using a large TCGA set, transferred its weights to the CCLE autoencoder as its weight initializations, and then extracted two-step pre-trained CCLE_enc_ to link to P. We trained the autoencoder using 90% of CCLE data together with 10% as the validation set. The optimal hyperparameters were searched by KerasTuner (https://keras-team.github.io/keras-tuner/) based on the loss of the validation set. As a result, for the architecture of encoder, the input layer had 5000 neurons, followed by one hidden layer with 512 neurons, and a bottleneck layer with 200 neurons. The activation function was set as LeakyReLU (alpha = 0.1) for each layer in the autoencoder. The complete algorithm for two-step pre-training CCLE_enc_ is given in Algorithm 1.


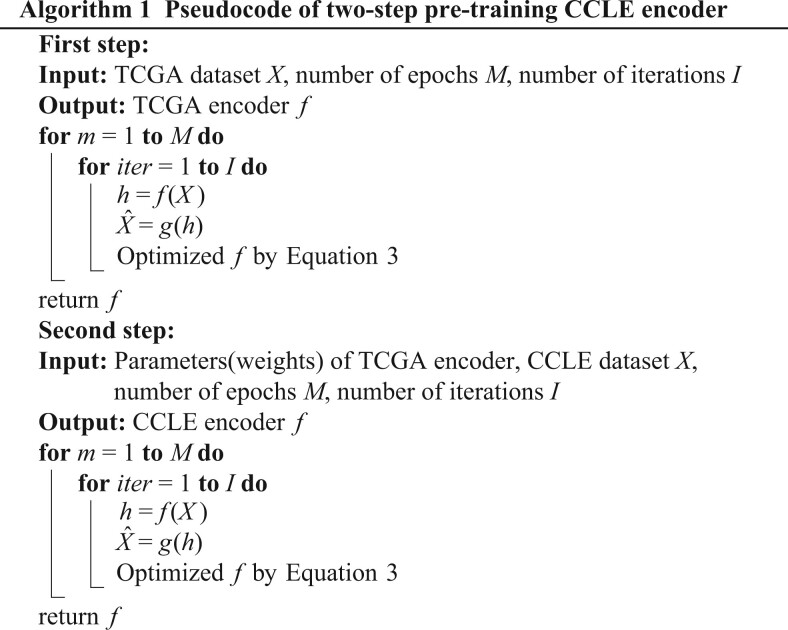


### Prediction network

Following the two-step pre-trained CCLE_enc_ is P, a 4-layer feedforward neural network, including the first layer merging output neurons of the two-step pre-trained CCLE_enc_, two fully connected layers, and one output layer generating predicted values. The architecture of two-step pre-trained CCLE_enc_ was fixed. Its initialized parameters came from two-step pre-training and the weights would be updated during the training process. P used default initialization. To enable various partitions of data as required by the different prediction scenarios and to obtain robust evaluation metrics including mean squared error (MSE), root mean squared error (RMSE), and Spearman rank correlation, we used the average value of five-fold cross-validation results for model evaluation. For each time, four-fold of data randomly split from the whole data were used together with 10% validation set and one-fold of data was used as a testing set. The architectures for the measurement types are detailed in File S1 and Table S1.

### Feature selection and model comparison

TransCell obtained compressed CCLE representation from the encoder. Except for using encoder capturing essential feature information, we investigated the common feature selection and dimension reduction methods, including least absolute shrinkage and selection operator (LASSO), elastic net (EN), random forest (RF), and principal component analysis (PCA) (File S1). The performance of TransCell was subsequently compared with that of the three baseline machining learning methods, including LASSO, EN, and RF, as well as two DNN designs (File S1). The average value [with 95% confidence interval (CI)] of five-fold cross-validation results measured by MSE and RMSE was used for model evaluation (File S1). During the preliminary stage, we used a random sample of *n* = 20 to assist us in selecting the appropriate model. Based on the evaluation metrics, we selected the model with the best performance and used it to make predictions at scale. We observed that the comparison results of model performance were consistent as *n* was increased to 200 or more (Table S2).

### Pediatric cancer sensitivity prediction

The gene expression profiles of pediatric cancer cell lines were downloaded from [36] and then fed into trained models. Drug sensitivity data were collected from PubChem Bioassay (RDES: AID1259252; TC32: AID1259256; EW8: AID1259255). PubChem Identifier Exchange Service was used to convert the simplified molecular input line entry system (SMILES) in DepMap to PubChem Compound Identification, which was later used to map drug sensitivity. PUBCHEM_ACTIVITY_OUTCOME was compared with predicted drug sensitivity. We considered predicted score < −2 as active compounds (a few other thresholds were explored as well). Fisher exact test was applied to identify selective compounds for each cancer. R packages umap and ComplexHeatmap were used to visualize cell lines and drug sensitivity.

## Results

We developed a pipeline to systematically evaluate models in the prediction of protein expression, CNV, mutation, metabolite, gene effect score, and drug sensitivity using gene expression data from CCLE (Figure 1B and C). The gene expression matrix consists of 1210 cell lines and 19,144 genes, while the 6 measurement types have a varying number of cell lines and measurements (Figure 1B). All these measurements are continuous except mutation, a binary measurement. For each measurement type, we started with a small set of measurements for the following models: LASSO, EN, RF, PCA features + DNN, and DNN + default initialization (see Method for details). For mutation data, we considered Logistic_LASSO and Logistic_EN. Each model comprises feature selection and regression/classification (Figure 1B). The best available model, chosen based on the evaluation metrics, was then adopted to perform the scale-up prediction. Due to the constraint of computation power, we randomly selected 2000 measurements for each type if the total measurements exceeded 2000. Moreover, gene enrichment analyses of the well and poorly predicted measurements were performed using the R package clusterProfiler (Figure 1C) [37].

We further developed TransCell, which is a framework for predicting molecular measurements by utilizing the transfer learning technique. TransCell first builds an autoencoder using a larger dataset comprising about 10,000 gene expression profiles from TCGA. The parameters of the resulting TCGA encoder are used to initialize the autoencoder built from CCLE gene expression profiles. Most transfer learning (or domain adaptation) methods align the source and target domains by creating a domain-invariant feature representation which requires the features to follow the same distribution. In light of the difference between tumors and cell lines, TransCell only encodes the genes sharing a similar distribution between the two sets. Subsequently, a two-step pre-trained CCLE_enc_ is extracted and then linked to a feedforward prediction network P.

### Metabolite prediction

**Figure 2:**
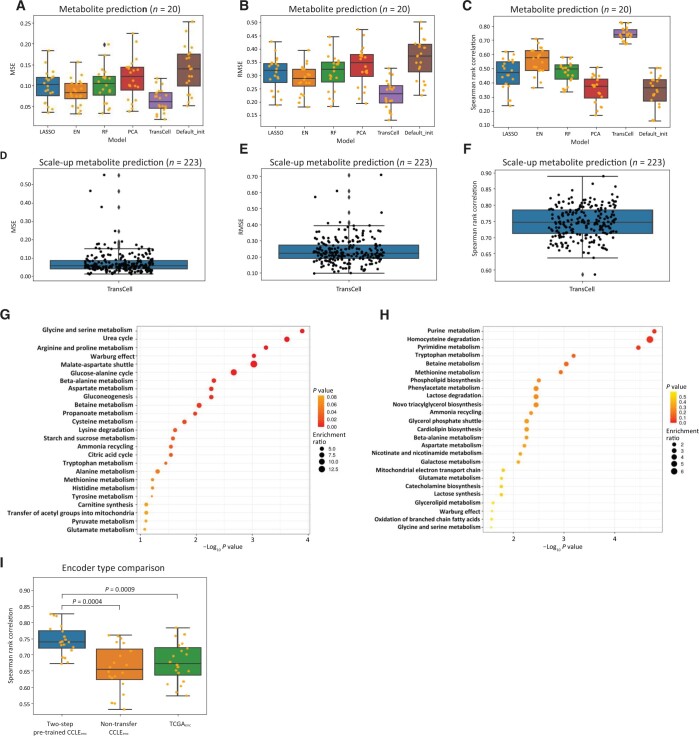
Metabolite prediction **A**.–**C**. The boxplots of MSE (A), RMSE (B), and Spearman rank correlation (C) for different models, including LASSO, EN, RF, TransCell, and two DNN designs with PCA and Default_init in 20 metabolite prediction models. **D**.–**F**. The boxplots of MSE (D), RMSE (E), and Spearman rank correlation (F) for TransCell in all 223 scale-up metabolite prediction models. **G**. Enrichment analysis for metabolites with RMSE lower than the first quartile (well predicted metabolites) in all 223 scale-up metabolite predictions. **H**. Enrichment analysis for metabolites with RMSE greater than the third quartile (poorly predicted metabolites) in all 223 scale-up metabolite predictions. **I**. Spearman rank correlation for the TransCell architecture using two-step pre-trained CCLE_enc_, non-transfer CCLE_enc_, and TCGA_enc_. MSE, mean squared error; RMSE, root mean squared error; Default_init, default initialization; TCGA_enc_, TCGA encoder.

Metabolomics analyses facilitate the identification of biomarkers and improve the understanding of biological pathways in health and disease. Among the six molecular types in DepMap, metabolomics is the last one becoming publicly available [31]. Its prediction using gene expression is barely explored, thus we first started with metabolite prediction. In order to quickly sense which model has the best performance, we randomly chose 20 metabolites to build a model for each. After removing missing values, 915 cell lines were left for prediction. A model was built for each metabolite individually. Among the predictions of 20 metabolites, TransCell reached the average values of MSE and RMSE 0.064 [95% CI: 0.051–0.075] and 0.228 (95% CI: 0.204–0.252), respectively, which were lower than those of LASSO, EN, and RF (**Figure 2**A and B; Table S3). Furthermore, TransCell outperformed DNNs with PCA or default initialization. The average value of Spearman rank correlation computed between true values and predicted values among 20 models was 0.744 (95% CI: 0.724–0.764) in TransCell, which was substantially higher than other models (Figure 2C). The evaluation metrics and Spearman rank correlation suggest that TransCell has the best performance in metabolite prediction. Therefore, we adopted TransCell to predict all 225 metabolites. In the scale-up prediction, the average values of MSE, RMSE, and Spearman rank correlation were 0.072 (95% CI: 0.064–0.080), 0.237 (95% CI: 0.226–0.247), and 0.746 (95% CI: 0.740–0.753), respectively (Figure 2D–F). Among those metabolites, the top 5 well predicted metabolites were C34:1 PC, C36:2 PC, C16:1 SM, C34:2 PC, and C38:4 PC. The top 5 poorly predicted metabolites were acetylcholine, taurochenodeoxycholate, methylnicotinamide, hypoxanthine, and palmitoylcarnitine.

To investigate well and poorly predicted metabolites, we derived those with RMSE lower than the first quartile as well predicted metabolites, and those with RMSE greater than the third quartile as poorly predicted metabolites. After that, we performed metabolite enrichment analyses using MetaboAnalyst [38]. The enrichment showed that the top 3 pathways related to well predicted metabolites were glycine and serine metabolism, urea cycle, and arginine and proline metabolism (Figure 2G). The pathways involved in purine metabolism, homocysteine degradation, and pyrimidine metabolism were related to poorly predicted metabolites (Figure 2H).

To demonstrate the efficacy of the two-step pre-trained CCLE_enc_, we explored another two types of encoders. The first type of encoder, CCLE_enc_, was trained by CCLE without transferred encoder parameters learned from TCGA. The second type of encoder, TCGA encoder (TCGA_enc_), was trained by TCGA. We repeated the analysis using the results from each new encoder. We found that TransCell using the two-step pre-trained CCLE_enc_ has the best performance (*P* < 0.05 using Wilcoxon rank-sum test) (Figure 2I), indicating that TransCell offers better parameter weight initializations.

### Gene effect score prediction

**Figure 3:**
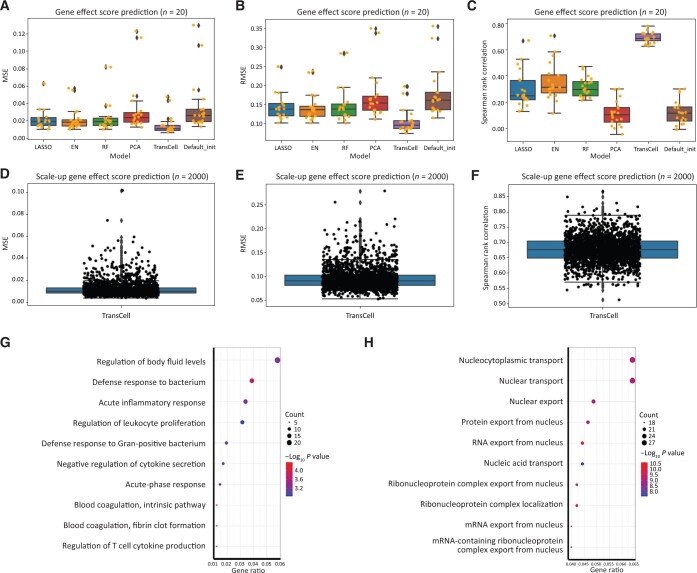
Gene effect score prediction **A**.–**C**. The boxplots of MSE (A), RMSE (B), and Spearman rank correlation (C) for different models, including LASSO, EN, RF, TransCell, and the two DNN designs with PCA and Default_init in 20 gene effect score prediction models. **D**.–**F**. The boxplots of MSE (D), RMSE (E), and Spearman rank correlation (F) for TransCell in 2000 scale-up gene effect score prediction models. **G**. and **H**. Enrichment analyses of biological processes for genes with RMSE lower than the first quartile (well predicted genes) (G) and higher than the third quartile (poorly predicted genes) (H) in the scale-up gene effect score predictions.

Similar to metabolite prediction, we first randomly chose 20 genes to build gene effect score prediction models. After removing missing values, 578 common cell lines between CCLE and DepMap were left. Among 20 gene effect score models built by TransCell, the average values of MSE and RMSE were 0.015 (95% CI: 0.010–0.020) and 0.105 (95% CI: 0.091–0.119), respectively, and the average value of Spearman rank correlation was 0.689 (95% CI: 0.672–0.707) (**Figure 3**A–C; Table S4). Among all the models, TransCell had the lowest MSE and RMSE and held the lowest variation and the highest Spearman rank correlation (*P* < 0.05, Student’s *t*-test), suggesting the superiority of TransCell in gene effect score prediction. Therefore, we used TransCell to predict 2000 randomly selected genes. The average values of MSE, RMSE, and Spearman rank correlation within these 2000 models were 0.012 (95% CI: 0.011–0.012), 0.095 (95% CI: 0.094–0.096), and 0.676 (95% CI: 0.675–0.678) (Figure 3D–F), respectively, corroborating with the small-scale test.

Based on RMSE distribution, we further examined well and poorly predicted genes (well: smaller than the first quartile; poorly: greater than the third quartile). Gene enrichment analyses found the top 3 pathways related to well predicted genes: (1) regulation of body fluid levels, (2) defense response to bacterium, and (3) acute inflammatory response (Figure 3G), and the top 3 pathways related to poorly predicted genes: (1) nucleocytoplasmic transport, (2) nuclear transport, and (3) nuclear export (Figure 3H). The poor performance of transporter genes might suggest the irrelevance of gene expression in predicting transporter function.

### Drug sensitivity prediction

**Figure 4:**
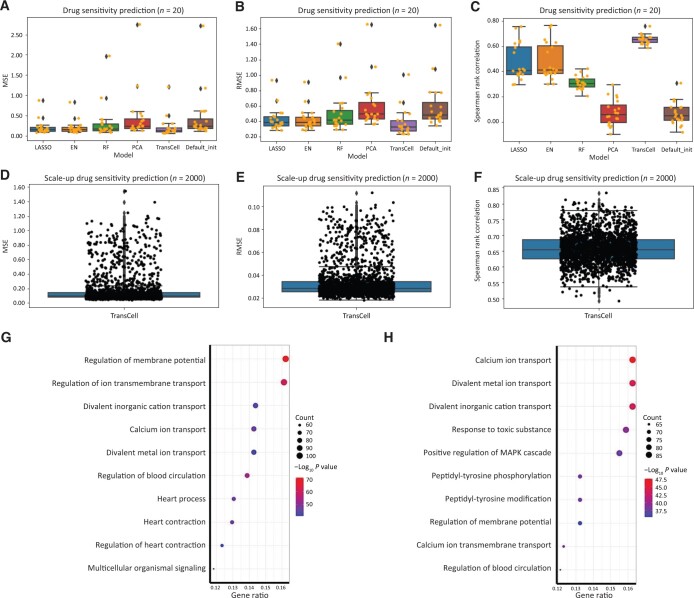
Drug sensitivity prediction **A**.–**C**. The boxplots of MSE (A), RMSE (B), and Spearman rank correlation (C) for different models, including LASSO, EN, RF, TransCell, and two DNN designs with PCA and Default_init in 20 drug sensitivity prediction models. **D**.–**F**. The boxplots of MSE (D), RMSE (E), and Spearman rank correlation (F) for TransCell in 2000 scale-up drug sensitivity prediction models. **G**. and **H**. Enrichment analyses of biological processes for target genes of compounds with RMSE lower than the first quartile (well predicted compounds) (G) and higher than the third quartile (poorly predicted compounds) (H) in the scale-up drug sensitivity predictions.

In drug sensitivity prediction, TransCell again outperformed others with the average values of MSE, RMSE, and Spearman rank correlation being 0.207 (95% CI: 0.094–0.320), 0.375 (95% CI: 0.295–0.455), and 0.653 (95% CI: 0.637–0.669), respectively (**Figure 4**A–C; Table S5). In the following scale-up drug sensitivity prediction, the average values of MSE, RMSE, and Spearman rank correlation within these 2000 models were 0.171 (95% CI: 0.162–0.180), 0.341 (95% CI: 0.334–0.348), and 0.657 (95% CI: 0.655–0.659), respectively (Figure 4D–F). One recent study using RF achieved the Pearson correlation of 0.389, which is close to our observation (Figure 4C) [26]. TransCell improved the correlation by over 50%. We next selected well and poorly predicted compounds and mapped them to their putative targets through the drug–target mappings provided in DepMap. Pathway enrichment analyses of these targets revealed that sensitivities of the compounds involved in the regulation of membrane potential, regulation of ion transmembrane transport, and divalent inorganic cation transport were accurately predicted, while those targeting calcium ion transport, divalent metal ion transport, and divalent inorganic cation transport were poorly predicted (Figure 4G and H).

### Protein, CNV, and mutation predictions

**Figure 5:**
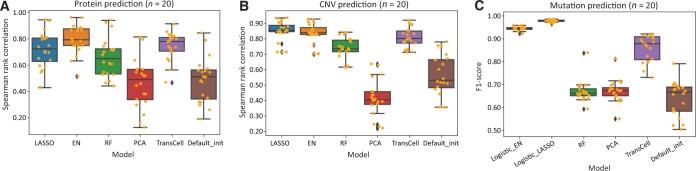
Protein, CNV, and mutation predictions **A**. and **B**. Performance of different models on protein prediction (A) and CNV prediction (B) evaluated by Spearman rank correlation. **C**. Performance of different models on mutation prediction evaluated by F1-score. Full prediction performance is shown in Figures S1–S3. CNV, copy number variation.

Unlike the previous three measurement types where TransCell performed the best, protein prediction tended to favor linear-based models like EN, which had the best performance with a Spearman rank correlation of 0.794 (95% CI: 0.745–0.843) (**Figure 5**A, Figure S1A–C; Tables S6–S8). The performance of TransCell was comparable, with no significant difference detected (Spearman rank correlation difference, Student’s *t*-test, *P* = 0.153). While scaling up to all 214 proteins using EN, the performance was stable [Spearman rank correlation = 0.792 (95% CI: 0.777–0.806)] (Figure S1D–F). Diving into individual proteins, we observed that the proteins related to apoptotic signaling pathway, regulation of protein complex assembly, and epithelial cell proliferation could be easily predicted. In contrast, those related to serine/threonine kinase activity, response to toxic substance, and response to nutrient levels were less likely to be inferred from gene expression (Figure S1G and H).

For investigating which proteins or group of proteins could be better predicted based solely on its encoding gene’s expression, we further conducted the experiments on the EN model for all proteins only based on their encoding genes. The average correlation reduced to 0.431 from 0.792, suggesting the importance of leveraging other genes’ expression in predicting protein abundance. According to the predicted results, we selected the proteins with Spearman rank correlation > 0.7 (computed by true values and predicted values) as well predicted proteins. After that, based on those proteins’ encoding genes, we performed gene enrichment analysis. The most corresponding biological processes were wound healing, gland development, and peptidyl-serine phosphorylation. Moreover, when we looked into individual top 4 well predicted proteins’ functions, they were involved in the regulation of innate and adaptive immunity, inflammatory response, cell proliferation, and cell migration (tyrosine-protein kinase SYK, annexin A1, epidermal growth factor receptor, and protein kinase C alpha type; all Spearman rank correlation > 0.9) (Table S9).

To find which genes could consistently be helpful in predicting the protein abundance of other proteins, we then computed the frequency of feature genes showing in all protein predictions based on the EN model. Among all protein predictions, if genes were used more than 60 times, we regarded them as helpful feature genes in making prediction of protein abundance and identified a total of 68 genes. Furthermore, those genes were related to response to xenobiotic stimulus, cellular response to peptide, and response to toxic substance. Notably, *GTSF1* and *MT1E* were used more than 100 times in protein abundance predictions, but it should be noted that the results could vary depending on the list of target proteins (Table S10).

Similar to protein expression, CNV can be easily predicted by linear models. LASSO stood out, with the average values of MSE, RMSE, and Spearman rank correlation being 0.009 (95% CI: 0.006–0.012), 0.087 (95% CI: 0.070–0.103), and 0.844 (95% CI: 0.814–0.874), respectively (Figure 5B, Figure S2A–C; Table S9). TransCell and EN had comparable performance with LASSO (*P* > 0.01). Subsequent modeling of 2000 genes using LASSO suggests the consistent power of predicting CNV using gene expression alone [Spearman rank correlation = 0.874 (95% CI: 0.872–0.876)] (Figure S2D–F). For the handful of poorly predicted genes, no prominent patterns were observed (Figure S2G and H).

In mutation prediction, we used the area under curve (AUC) and F1-score to evaluate the classification of mutation status (mutated/wild). Logistic_LASSO and Logistic_EN had the best performance, followed by TransCell. Among 20 mutation prediction models built by Logistic_LASSO, the average values of AUC and F1-score were 0.996 (95% CI: 0.995–0.997) and 0.978 (95% CI: 0.976–0.980), respectively, suggesting that mutation could be precisely predicted by gene expression (Figure 5C, Figure S3A and B; Table S10). While scaling up to 2000 mutations using Logistic_LASSO, nearly all the mutations could be well predicted (Figure S3C and D) [AUC = 0.990 (95% CI: 0.990–0.990); F1-score = 0.971 (95% CI: 0.970–0.971)]. In order to explore poorly predicted genes in the scale-up mutation prediction, we considered genes with AUC lower than the first quartile as poorly predicted. Afterward, we performed gene enrichment analyses of the biological process for those genes. The top 3 pathways ranked by gene ratio were (1) cell morphogenesis involved in neuron differentiation, (2) synapse organization, and (3) axon development (Figure S3E).

### Model evaluation using external and prospective datasets

Having shown the feasibility of using gene expression to predict other molecular measurements, mainly using DepMap, we next sought external validation to verify the robustness of the models. We realized the datasets with both RNA-seq and other measurements are surprisingly scarce; we could only explore a few datasets. First, we analyzed the proteomic data in CellMinerCDB (https://discover.nci.nih.gov/cellminercdb). The protein array data in CellMinerCDB were taken from MD Anderson Cell Lines Project (https://tcpaportal.org/mclp), and the RNA-seq data were based on the NCI60 cell lines. There were 60 cell lines and 36 common proteins between CellMinerCDB and DepMap. Among 36 protein prediction models built by EN, the average value of Spearman rank correlation was 0.360 (95% CI: 0.281–0.439) for external validation, and the prediction of 75% of the proteins was significantly correlated with the real expression (*P* < 0.05) (Tables S11 and S12). In general, a model with a better performance in the training set led to a better prediction in the external set (Spearman rank correlation = 0.48, *P* < 0.01). While excluding phosphorylated/cleaved proteins, the correlation could reach 0.42. Interestingly, proteins including alpha-catenin, N-cadherin, beta-catenin, and p53_Caution could achieve remarkable performance (Spearman rank correlation > 0.7). We further inspected the top poorly predicted proteins, estrogen receptor-alpha (ER-alpha) and progesterone receptor (PR), and found the correlations of their actual expression between DepMap and CellMinerCDB were only 0.035 and 0.25, respectively. The undesired performance reinforces the importance of high-quality data for both training and testing. In addition, the slight decrease of predictive performance is likely due to the batch effect of gene expression profiles and the lack of optimal normalization of the external data in the model. Nevertheless, under these various challenges, the independent external validation for the models of those shared proteins confirms their reasonable robustness.

**Figure 6:**
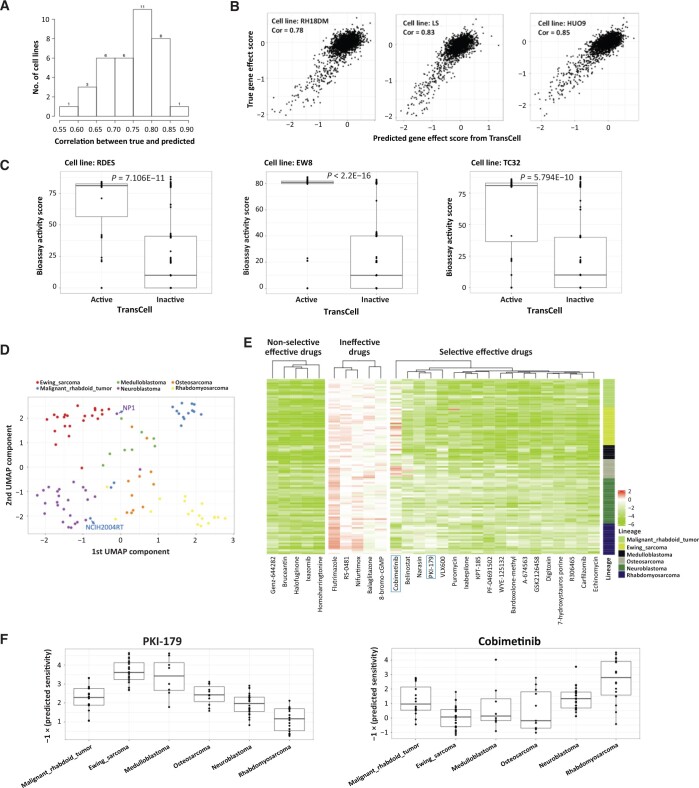
*In silico* expanding measurements for pediatric cell lines **A**. Distribution of correlations between predicted and true gene effect scores across 36 new cell lines. **B**. Scattering plots of predicted and true gene effect scores in three new pediatric cell lines. **C**. Validation of TransCell drug sensitivity prediction using published screening results in three pediatric cell lines. Active was defined as TransCell score < −2. The active score provided in PubChem Bioassay was adopted for comparison, and the two-tailed Student’s *t*-test was used to compute the difference. **D**. UMAP visualization of 101 pediatric cell lines based on predicted sensitivity of 2356 drugs. Only the cancer with at least ten cell line profiles was selected. **E**. Heatmaps of predicted sensitivity of non-selective effective drugs, ineffective drugs, and selective effective drugs. Non-selective drugs stand for the drugs which are highly effective in the majority of cell lines (TransCell score < −2); ineffective drugs stand for the drugs which are ineffective in the majority of cell lines (TransCell score > −2); selective effective drugs stand for the drugs which are only effective in a specific cancer. For each cancer, a Fisher’s exact test was used to compute compound selectivity. Only five non-selective drugs and five ineffective drugs were visualized. **F**. Example of two selective drugs PK1-179 and cobimetinib. UMAP, Uniform Manifold Approximation and Projection.

Next, we turned to the recent DepMap 20Q1 release to explore if the model is applicable to prospective data. We found 36 new cell lines with both gene expression and gene effect scores. For each cell line, we used TransCell to predict the effect score of 2000 genes. The predicted effect scores were significantly correlated with the real effect scores, with over 70% of cell lines presenting a high Spearman rank correlation of > 0.7 (**Figure 6**A). Besides, we investigated three new pediatric cancer cell lines. As expected, all of them had a correlation higher than 0.7 (Figure 6B). Given the finding that the correlation of all the gene scores across all genes and cell lines between two independent experimental CRISPR-Cas9 screening studies could only achieve 0.7 [39], the remarkable performance of TransCell warrants its wide application in other cell lines.

### Case 1: *in silico* drug screening for pediatric cancer cell lines identifies new repurposing candidates

We implemented TransCell to infer the sensitivity of 4686 DepMap drugs for the 124 pediatric cell lines recently published (Table S13) [36]. We started with evaluating the performance in three Ewing sarcoma cell lines (RDES, EW8, and TC32), for which we could find the screening results in PubChem Bioassay. The sensitivity data of 305 drugs in each cell line were retrieved from PubChem. As expected, the active compounds predicted by TransCell (score < −2) had much higher activities than inactive compounds in all cell lines (Figure 6C), further confirming its reasonable performance. Next, we clustered all the cell lines of six common pediatric cancers, and found that the predicted drug sensitivity could further classify cancer types (Figure 6D). Interestingly, we observed that some cell lines had very different responses from most of the cell lines with the same origin. For example, NB1 (neuroblastoma cell line) and NCIH2004RT (malignant rhabdoid tumor) more closely resembled Ewing sarcoma and neuroblastoma, respectively. We further investigated individual drugs. In total, 29 drugs including homoharringtonine and ixazomib were effective in over 90% of pediatric cancer cell lines (Figure 6E). Notably, a number of drugs were only effective in specific cancers (Figure 6E). For instance, Ewing sarcoma and medulloblastoma cells were more sensitive to PKI-179, a phosphoinositide-3-kinase/mammalian target of rapamycin (PI3K/mTOR) inhibitor, while rhabdomyosarcoma cells were more sensitive to cobimetinib, a mitogen-activated protein kinase (MEK) inhibitor (Figure 6F). The prevalence of selective compounds implicates the potential of leveraging the predicted profiles to identify novel repurposing candidates and their biomarkers.

### Case 2: *in silico* CRISPR-Cas9 knock-out captures drug resistance in melanoma

**Figure 7:**
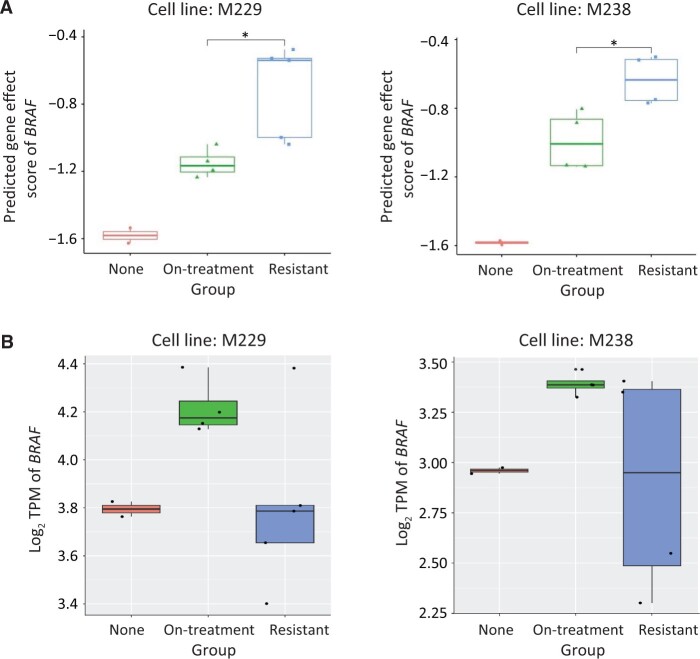
*In silico* CRISPR-Cas9 knock-out captures drug resistances **A**. Predicted gene effect scores of *BRAF* in two melanoma cell lines M229 and M238. RNA-seq profiles downloaded from GEO (GSE75299) were processed using the OCTAD pipeline (see Method for details). A lower score means a high gene dependency (DepMap used −1 as a threshold to define the dependency). A two-sided Student’s *t*-test was used to compute the difference. Note that the top sample in the resistant group in M229 was treated by the combination of BRAFi and MEKi. **B**. Expression level of *BRAF* for each sample among none, on-treatment, and resistant groups in M229 and M238 cell lines, respectively. GEO, Gene Expression Omnibus; TPM, transcript per million; BRAFi, BRAF inhibitor; MEKi, MEK inhibitor.

Using melanoma-resistant cell lines as an example, we demonstrated that TransCell could efficiently provide *in silico* measurement of modified cell lines. To understand dynamic transcriptomic states, Song et al. [40] profiled parental cell lines (including M229 and M238) treated with dimethyl sulfoxide (DMSO/vehicle), on-treatment lines [days to weeks on BRAF inhibitor (BRAFi)], and resistant lines [months to years on BRAFi or BRAFi + MEK inhibitor (MEKi)]. Using their gene expression profiles [Gene Expression Omnibus (GEO): GSE75299], we deployed TransCell to predict the *BRAF* gene effect score. It is known that the cells treated with BRAFi become less dependent on *BRAF*. TransCell could nicely capture the difference of *BRAF* dependencies among three groups (none, on-treatment, resistant) in both M229 and M238 (**Figure 7**A). The predicted scores of our non-resistant lines were close to the scores in the majority of skin cancer cell lines [5] and the predicted scores of the resistant M229 line were similar to the scores of another resistant M229 line for which our lab profiled recently for drug discovery [41] (the predicted scores of the first three resistant samples in GEO: GSE145990 were −0.58, −0.54, −0.52, respectively). Although these experiments were conducted in different labs, the predicted scores were very consistent. Further, the gene effect score was not associated with the *BRAF* expression level in M229 and M238, suggesting that genetic dependency is dependent on the expression of other genes (Figure 7B). Together, we show that TransCell gene effect score prediction could be a proximity of the CRISPR-Cas9 knock-out experiment when a genome-wide knock-out experiment is not feasible to perform.

### TransCell: a web portal to predict six measurement types based solely on gene expression profiles

To make TransCell available to the community, we developed TransCell into a R–Shiny web portal that allows researchers predicting six measurement types including metabolite, protein, CNV, gene effect score, drug sensitivity, and mutation given gene expression profiles. We trained one model for each individual feature, and the optimal hyperparameters were searched through KerasTuner based on one randomly chosen model and then applied to all individual features for each type of prediction. Furthermore, for each individual feature, in order to obtain robust results, we saved five models based on five-fold cross-validation. TransCell later would utilize them to provide the average predicted result. In total, we precomputed 352,740 models: [225 (the number of metabolites) + 214 (the number of proteins) + 18,333 (the number of genes in gene effect score dataset) + 4686 (the number of drugs in drug sensitivity dataset) + 27,639 (the number of genes in CNV dataset) + 19,451 (the number of genes in mutation dataset)] × 5 = 352,740. These models required approximately 4 terabytes of storage. The training time of five-fold cross-validation for each individual feature was about 5 min on an Amazon Web Services server (g3.4xlarge). All the models were processed by GNU parallel computation [42]. TransCell web portal only provides 15,010 models whose macro F1-scores are greater than 0.7 in mutation prediction. Users could upload new gene expression profiles [log_2_ (1 + TPM)] and select their interested features for making predictions. After TransCell computation is finished, users can either download the results from the portal or can receive the results through email if valid email address is provided.

## Discussion

Gene expression is arguably the most widely used modality in biomedical research. GEO, the largest expression repository, has archived 42 million profiles as of Jan, 2021, and the number continues to multiply. The advances of single-cell technology even enable the generation of thousands of expression profiles in a single experiment. Gene expression profiling of new samples today becomes routine. Compared with gene expression, other modalities, especially those large-scale perturbation experiments, are not easy, if not impossible, to produce in most labs. If gene expression could be leveraged to predict other modalities, additional lenses could be added to gain more biological insights. It is known that gene expression encodes the information of other modalities, but whether the baseline models and existing data could decode such relations remains unknown. To our best knowledge, this work is one of the first to provide a comprehensive study of molecular measurement prediction using gene expression data. We also developed TransCell, which utilizes the transfer learning technique to gain better weight initializations learned from a larger dataset TCGA, for improving the performance of a model.

Except for genes with data imbalanced issues in mutation prediction, the easiness of predicting six measurement types is ranked as follows: mutation, CNV, protein, metabolite, gene effect score, and drug sensitivity. The top performance of mutation, CNV, and protein levels could reflect their inherent relationship that genetic variation regulates gene expression, which controls protein abundance. Metabolite abundance is directly related to protein expression rather than gene expression, and its abundance is very dynamic; however, surprisingly, the majority of metabolites can be fairly predicted using TransCell. The inferior performance of perturbation experiments (CRISPR-Cas9 knock-out and drug treatments) confirms that the complicated cellular responses could not be easily captured by linear models. Besides, the quality of the high-throughput experiments sets back model development [43]. TransCell adequately addresses such challenges. Notably, TransCell performs reasonably well in prospective datasets as well as external datasets. Two case studies further suggest the potential of using TransCell in biomedical research.

Although we have demonstrated the feasibility of using gene expression to predict other measurements, the following work could be extended in the future. First, the meta data of cell lines and tumors including age, gender, and disease lineage could contribute to model performance. We conducted experiments in which commonly used meta data including age, gender, and disease lineage were added as features in the metabolite prediction; we did not observe any significant improvement (Figures S4 and S5; File S1). Other than the original feature gene selection method for TransCell, we further conducted feature gene selection by considering immune genes, stromal genes [44], housekeeping genes [45], and overexpressed genes [46] of four common cancers including lung, skin, breast, and ovary cancers between CCLE and TCGA; we found that two feature gene selection methods showed similar results (Figure S6; File S1), suggesting that TransCell captures the essential features accounting for cellular responses and is robust to noisy features. Even though some meta data might be informative in some tasks, the scarcity of meta data associated with gene expression profiles limits its power in practice.

Second, more extensive evaluation of TransCell using external profiles is highly desired to confirm the robustness of this approach. In particular, due to the differences between cell lines and tissues [47,48], additional care should be taken while applied to those samples different from cancer cell lines. For instance, in metabolite prediction, while the steady-state assumption is a useful simplification in many cases, it is not always accurate, especially in dynamic biological systems; therefore, before applying predictive models to real tumor microenvironment (TME), further validations have to be conducted. Interestingly, in additional exploration of using the TransCell concept to predict clinical responses, we observed that a two-step deep transfer learning model performed better than that without transfer learning (Figure S7; File S1).

Third, the performance of TransCell in the prediction of complicated measurements such as metabolite could be improved through further tuning and integration with more biological data such as protein–metabolite and protein–protein interactions. We used KerasTuner to search for the optimal hyperparameters. While interpreting individual performances in poorly predicted measurements is challenging, a large-scale comparison of various hyperparameters in individual tasks, and the incorporation of metabolite relations in the model could help improve the performance. We preliminarily evaluated a multi-task learning model for metabolite prediction based on TransCell’s architecture, and observed that sharing information with unrelated tasks might result in negative transfer (Figures S8 and S9; File S1). Given the nature of the metabolic profiling methods, the abundance of different metabolites cannot be directly compared [31], and thus additional care is needed while attempting to leverage their relations in modeling.

Lastly, different from many published studies where additional omics data other than gene expression such as chemical structures and mutations were incorporated in order to improve performance [49], this work aims to answer if it is feasible to use gene expression alone to predict other measurement types. In the potential applications, acquiring additional data for new gene expression profiles is challenging, if not impossible, and thus our model comparison tends not to include other additional features. Due to the unique goal of this work, many state-of-the-art models that need additional features are not comparable to ours. Nevertheless, we indeed compared one relevant published model DeepDR [10] in drug sensitivity prediction and demonstrated the comparable performance of TransCell (Figure S10; File S1). We reason the difference of performance is due to the consideration of the features sharing the same distribution between TCGA and CCLE which could avoid the negative transfer. Moreover, since TransCell makes the prediction based solely on gene expression, it mitigates the quantity of uncertainties caused by transferring the information coming from different types of data as well compared with DeepDR. In short, this survey confirms that large-scale *in silico* characterization of genomic landscape and cellular responses from gene expression for new or modified cell lines is feasible and future research could include model interpretability and application.

## Code availability

The source codes required in this study are available in GitHub (https://github.com/Bin-Chen-Lab/transcell). Please refer to the following link for TransCell web portal (http://apps.octad.org/transcell/).

## CRediT author statement

**Shan-Ju Yeh:** Conceptualization, Methodology, Formal analysis, Software, Validation, Data curation, Visualization, Writing – original draft, Writing – review & editing. **Shreya Paithankar:** Software, Validation, Data curation, Visualization. **Ruoqiao Chen:** Validation, Visualization. **Jing Xing:** Writing – review & editing. **Mengying Sun:** Writing – review & editing. **Ke Liu:** Software, Writing – review & editing. **Jiayu Zhou:** Writing – review & editing. **Bin Chen:** Conceptualization, Investigation, Writing – review & editing, Supervision, Project administration, Funding acquisition. All authors have read and approved the final manuscript.

## Supplementary material

Supplementary material is available at *Genomics, Proteomics & Bioinformatics* online (https://doi.org/10.1093/gpbjnl/qzad008).

## Competing interests

The authors have declared no competing interests.

## Supplementary Material

qzad008_Supplementary_Data
