# Supplementary material for: TransCell: In Silico Characterization of Genomic Landscape and Cellular Responses by Deep Transfer Learning
Source: Genomics Proteomics Bioinformatics. 2024 Sep 6;22(2):qzad008. doi: 10.1093/gpbjnl/qzad008 (PMC11378636; doi:10.1093/gpbjnl/qzad008)
Supplement: qzad008_Supplementary_Data [file qzad008_supplementary_data.zip › FigS5.pdf]

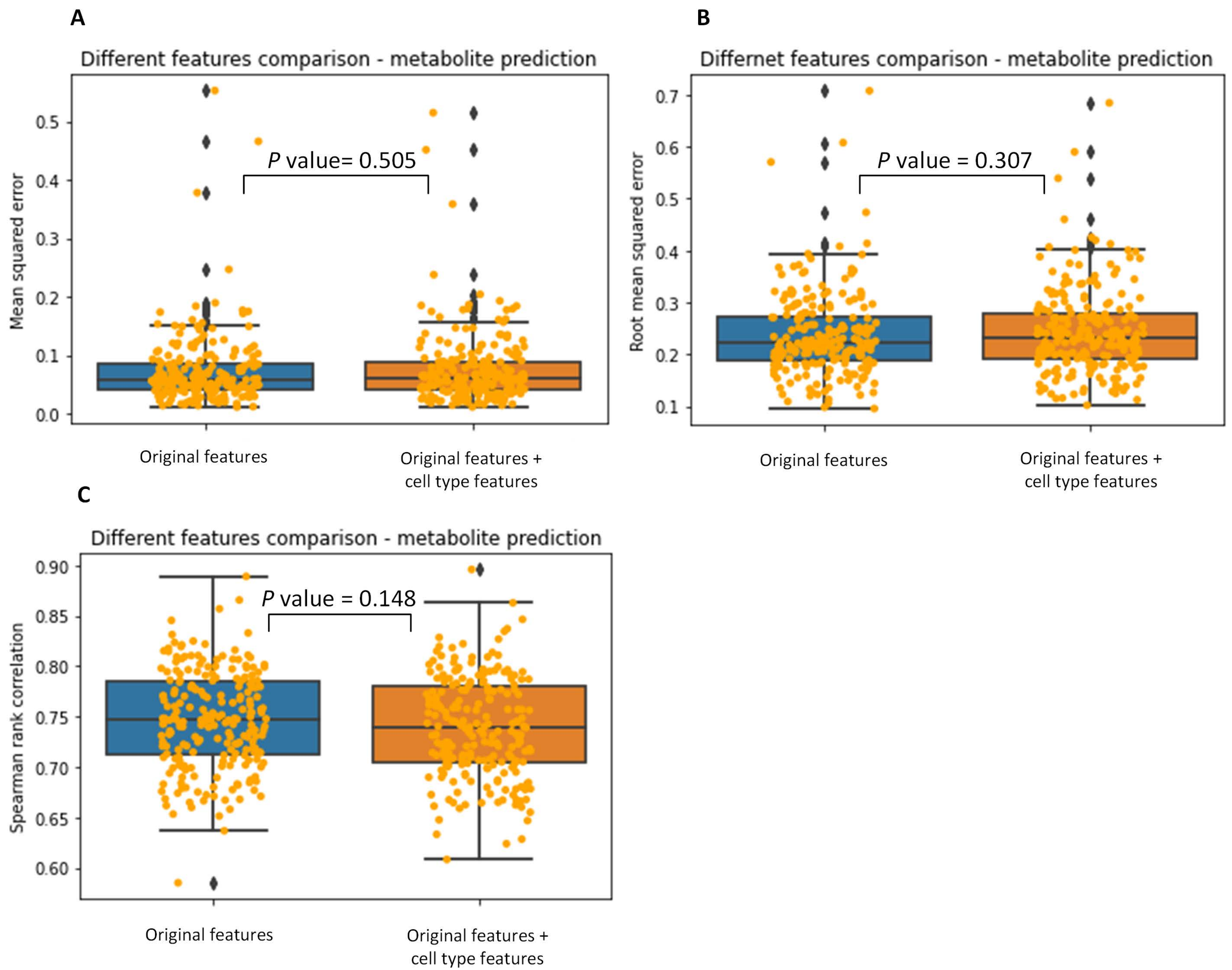
A
Different features comparison - metabolite prediction
P value= 0.505
Mean squared error
0.0
0.1
0.2
0.3
0.4
0.5
Original features
Original features +
cell type features
B
Differnet features comparison - metabolite prediction
P value = 0.307
Root mean squared error
0.1
0.2
0.3
0.4
0.5
0.6
0.7
Original features
Original features +
cell type features
C
Different features comparison - metabolite prediction
P value = 0.148
Spearman rank correlation
0.60
0.65
0.70
0.75
0.80
0.85
0.90
Original features
Original features +
cell type features
