# Supplementary material for: TransCell: In Silico Characterization of Genomic Landscape and Cellular Responses by Deep Transfer Learning
Source: Genomics Proteomics Bioinformatics. 2024 Sep 6;22(2):qzad008. doi: 10.1093/gpbjnl/qzad008 (PMC11378636; doi:10.1093/gpbjnl/qzad008)
Supplement: qzad008_Supplementary_Data [file qzad008_supplementary_data.zip › FigS6.pdf]

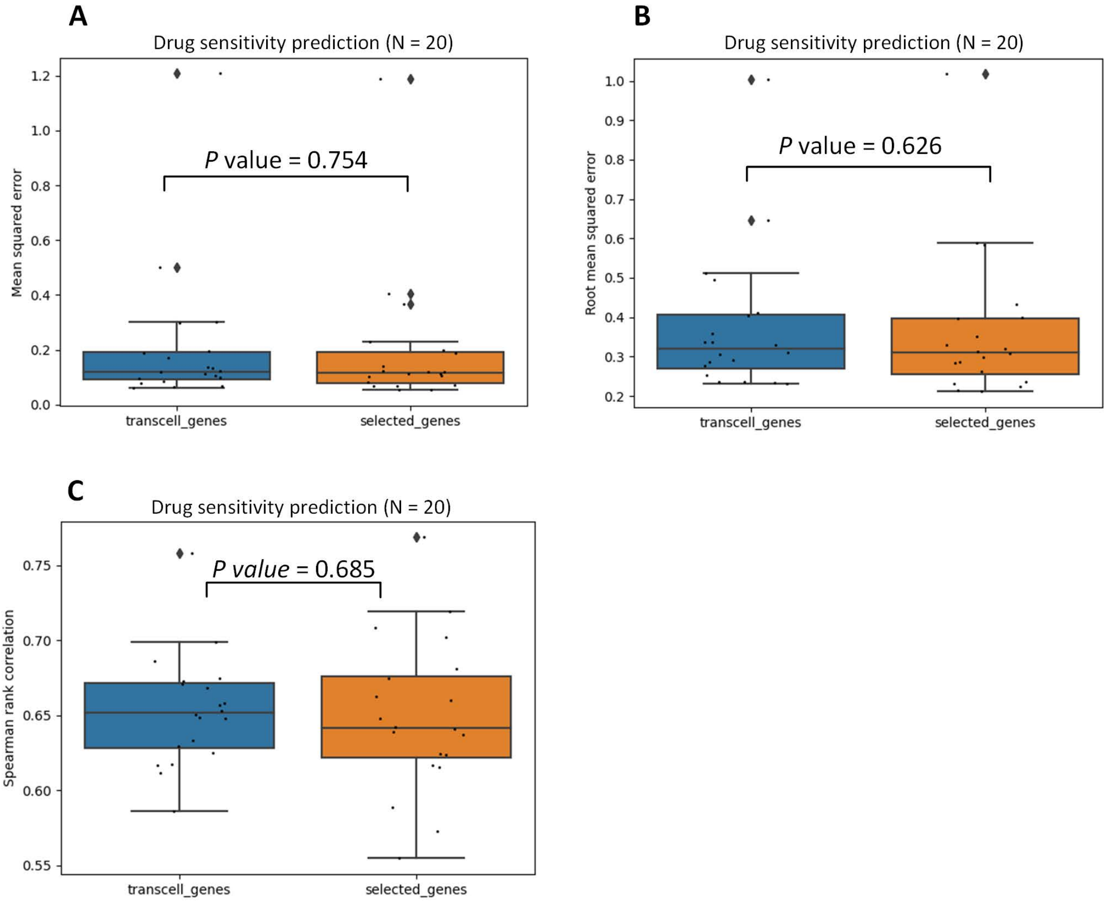

A
Drug sensitivity prediction (N = 20)
Mean squared error
P value = 0.754
0.0
0.2
0.4
0.6
0.8
1.0
1.2
transcell_genes
selected_genes
B
Drug sensitivity prediction (N = 20)
Root mean squared error
P value = 0.626
0.2
0.3
0.4
0.5
0.6
0.7
0.8
0.9
1.0
transcell_genes
selected_genes
C
Drug sensitivity prediction (N = 20)
Spearman rank correlation
P value = 0.685
0.55
0.60
0.65
0.70
0.75
transcell_genes
selected_genes
