# Supplementary material for: TransCell: In Silico Characterization of Genomic Landscape and Cellular Responses by Deep Transfer Learning
Source: Genomics Proteomics Bioinformatics. 2024 Sep 6;22(2):qzad008. doi: 10.1093/gpbjnl/qzad008 (PMC11378636; doi:10.1093/gpbjnl/qzad008)
Supplement: qzad008_Supplementary_Data [file qzad008_supplementary_data.zip › Supplementary material captions.docx]

## Supplementary material

**File S1 Supplementary text for TransCell**

**Figure S1 Protein predictions**

**A.**–**C.** The boxplots of (A) MSE, (B) RMSE, and (C) Spearman rank correlation for different predictors, including LASSO, EN, RF, TransCell, and two DNN designs with PCA and default initializations in 20 protein prediction models. **D.**–**F.** The boxplots of (D) MSE, (E) RMSE, and (F) Spearman rank correlation for EN in all 214 scale-up protein prediction models. **G.** and **H.** The dot plots of gene enrichment analysis of biological processes for target genes of proteins with RMSE (G) lower than the first quartile (well predicted proteins) and (H) higher than the third quartile (poorly predicted proteins) in the scale-up protein predictions.

**Figure S2 CNV predictions**

**A.**–**C.** The boxplots of (A) MSE, (B) RMSE, and (C) Spearman rank correlation for different predictors, including LASSO, EN, RF, TransCell, and two DNN designs with PCA and default initializations in 20 CNV prediction models. **D.**–**F.** The boxplots of (D) MSE, (E) RMSE, and (F) Spearman rank correlation for LASSO in 2000 scale-up CNV prediction models. **G.** and **H.** The dot plots of gene enrichment analysis of biological processes for genes with RMSE (G) lower than the first quartile (well predicted genes) and (H) higher than the third quartile (poorly predicted genes) in the scale-up CNV predictions.

**Figure S3** **Mutation predictions**

**A.** and **B.** The boxplots of (A) AUC and (B) F1 score for different predictors, including Logistic_EN, Logistic_LASSO, RF, TransCell, and two DNN designs with PCA and default initializations in 20 mutation prediction models. **C.** and **D.** The boxplot of (C) AUC and (D) F1 score for Logistic_LASSO in 2000 scale-up mutation prediction models. **E.** The dot plot of gene enrichment analysis of biological processes for genes with AUC lower than the first quartile (poorly predicted genes) in the scale-up mutation prediction. AUC, area under curve.

**Figure S4 Different feature set combination comparisons in all metabolite models based on the average of five-fold cross-validation results**

**A.**–**C.** The boxplots of (A) MSE, (B) RMSE, and (C) Spearman rank correlation.

**Figure S5 Different feature set combination comparisons in all metabolite models based on the average of five-fold cross-validation results**

**A.**–**C.** The boxplots of (A) MSE, (B) RMSE, and (C) Spearman rank correlation.

**Figure S6 Different feature gene selection methods in drug sensitivity prediction based on the average of five-fold cross-validation**

**A.**–**C.** The boxplots of (A) MSE, (B) RMSE, and (C) Spearman rank correlation.

**Figure S7 ROC for clinical prediction framework with pre-two-stage transfer learning and without transfer learning**

ROC, receiver operating characteristic.

**Figure S8 The architecture of multi-task learning model for metabolite predictions**

**Figure S9 The boxplot of root mean squared error between the same 20 metabolite predictions for model type comparison**

**Figure S10 Model comparisons between TransCell and DeepDR for cancer cell line (CCLE) drug response prediction based on the genomics of drug sensitivity in cancer (GDSC) project**

The boxplots show the root mean squared error in log-scale IC_50_ for TransCell and DeepDR, respectively. GDSC, Genomics of Drug Sensitivity in Cancer.

**Table S1 Searching space of hyperparameters for baseline machine learning methods**

**Table S2 Finding patterns of model evaluation for each measurement type prediction**

**Table S3 Metabolite prediction**

**Table S4 Gene effect score prediction**

**Table S5 Drug sensitivity prediction**

**Table S6 Protein prediction**

**Table S7 Copy number variation prediction**

**Table S8 Mutation prediction**

**Table S9 Protein prediction results based on their encoding genes under elastic net models**

**Table S10 Frequency of feature genes shown in protein predictions under elastic net models**

**Table S11 Internal validation of protein expression prediction**

**Table S12 External validation using CellMinerCDB protein array data**

**Table S13 *In silico* expansion of drug sensitivity for pediatric cancer cell lines**
