# Supplementary material for: TransCell: In Silico Characterization of Genomic Landscape and Cellular Responses by Deep Transfer Learning
Source: Genomics Proteomics Bioinformatics. 2024 Sep 6;22(2):qzad008. doi: 10.1093/gpbjnl/qzad008 (PMC11378636; doi:10.1093/gpbjnl/qzad008)
Supplement: qzad008_Supplementary_Data [file qzad008_supplementary_data.zip › Supplementary Text_0526-done_DEC.docx]

**File S1 Supplementary text for TransCell**

**Transfer learning**

The high dimensional gene expression features and limited cell lines pose challenges to any machine learning algorithms [1]. Prior studies show that autoencoder, an unsupervised artificial neural network that could capture nonlinear relationships between features, can learn a robust representation of gene expression data. We also observed the superiority of autoencoder in representing tumor gene expression profiles in reference tissue selection [2]. However, its performance highly depends on the initial parameters when the training set is small. One approach to overcome data scarcity is to apply transfer learning techniques. Transfer learning aims to improve the performance of target learners on target domains by transferring the knowledge learned from different but related source domains and then applying the knowledge to a target task [3,4]. The weights and architecture obtained from the pre-trained model could be directly used to initialize the target task [5]. Moreover, weight optimization in nonlinear autoencoders is cumbersome. With large initial weights, autoencoders typically find poor local minima. A pre-training procedure was introduced to provide an effective way of initializing the weights of deep autoencoder networks [6].

**Comparison to other model designs**

One primary reason to evaluate feature selection and dimension reduction methods, as well as predictors, is to choose the best available model and estimate how well a given model is likely to perform in making predictions for each measurement type. The performance of TransCell was compared to three baseline machining learning methods, including least absolute shrinkage and selection operator (LASSO), elastic net (EN), and random forest (RF), and two deep neural network (DNN) designs. Many existing algorithms were developed for individual tasks, and thus were not included for a back-to-back comparison, but we investigated some individually. The LASSO predictor relied on LASSO to perform feature selection and chose all the features with non-zero coefficient to be the essential features that were later selected to build a LASSO model. The EN predictor included an elastic net to identify all features with non-zero coefficients as essential features, followed by an elastic net model. The RF predictor used its internal feature selection method to build the model with feature importance > 10−4 as essential features.

We designed two similar DNN architectures for comparisons, with one replacing two-step pre-trained Cancer Cell Line Encyclopedia (CCLE) encoder (CCLEenc) with principal component analysis (PCA) (using 200 principal components instead of the 200 compressed autoencoder features) and another training with default initializations without transferred parameters. To conduct a performance comparison in mutation prediction, a logistic regression model was applied for both feature selection and classification. Note that we compared the logistic regression model with regularization (Logistic_LASSO) and the logistic regression model with the combination of and regularizations (Logistic_EN), two different ways of feature selection in logistic regression. To reduce the searching time of hyperparameters, for each type of predictor in the prediction for each measurement type, we derived hyperparameters based on one model and applied them to all models. The optimized hyperparameters are found by scikit-learn’s GridSearchCV for LASSO, EN, RF, Logistic_LASSO, and Logistic_EN. During cross-validation tuning, we used mean squared error (MSE) as a scoring metric for the best hyperparameters search. The actual values we used for feature selection and prediction model in each type of predictor are shown in Table S1. The computing time for each predictor to obtain an average of five-fold cross-validation results is around five min.

**Model evaluation**

MSE and root mean squared error (RMSE) are commonly used model evaluation metrics for measuring model performance. Both MSE and RMSE are sensitive to outliers, meaning that they give more weight to larger differences. Because of giving higher weight to unfavorable conditions, they usually are good at revealing model performance differences [7]. In order to make further intuitive assessments, we also computed Spearman rank correlation between predicted and actual values. It is noted that RMSE is not only interpretable in terms of measurement units but also a better measurement of model fit than a correlation coefficient [8]. Therefore, we used RMSE to derive well predicted features and poorly predicted features, upon which gene or metabolite set enrichment analyses of biological processes were performed. To avoid overfitting and biased results, the evaluation metrics were computed based on the average of five-fold cross-validation results. In the five-fold cross-validation, hyperparameters were fixed, and each of the k fold is given an opportunity to be used as a hold-out test set while all other k−1 folds are used as a training set. This procedure would repeat for k times iteratively. Therefore, we could compute the mean of evaluation metrics.

**The architecture of TransCell for metabolite prediction**

Here, we provide the detailed architecture of TransCell in metabolite prediction. The TransCell contains two-step pre-trained CCLEenc and a feed forward prediction network (P). P used Adam as an optimizer (learning rate = 0.001) with MSE loss. The input layer merging the output of two-step pre-trained CCLEenc had 352 neurons, followed by two hidden layers with 320 and 64 neurons. The activation function was set as a hyperbolic tangent for each layer. Moreover, to mitigate the overfitting problem, except for the output layer, the regularization penalties were applied to the output and bias for each layer. We used dropout 0.55, 0.60, and 0.50 on the input layer and two hidden layers, respectively. In addition, early stopping was used to terminate model training once the model performance stopped improving on the validation set. The optimal hyperparameters in TransCell were searched by the KerasTuner with 100 max trials. Before computing the evaluation metrics and Spearman rank correlation, we performed the inverse transformation on the predicted values, making them back to their original space without data rescaling by min–max scaler.

**The architecture of TransCell for gene effect score prediction**

In gene effect score prediction, the cancer cell line RNA sequencing (RNA-seq) transcript per million (TPM) gene expression data (CCLE) was rescaled in the range of 0 to 1 and the target data was rescaled in the range of −1 to 1 by min–max scaler. The TransCell contains two-step pre-trained CCLEenc and P. For the architecture of P, we used Adam as an optimizer (learning rate = 0.0009) with MSE loss. The input layer merging the output of two-step pre-trained CCLEenc had 288 neurons, followed by two hidden layers with 128 and 64 neurons. The activation function was set as a hyperbolic tangent for each layer. Moreover, to alleviate model suffering from overfitting, except for the output layer, we added regularization penalties on the output and bias for each layer. The dropouts were 0.60, 0.35, and 0.2 for the input layer and two hidden layers, respectively. In addition, early stopping was used to terminate model training once the mode performance stopped improving on the validation set. The optimal hyperparameters in the TransCell were searched by KerasTuner with 100 max trails. Before computing the evaluation metrics and Spearman rank correlation, we performed an inverse transform on the predicted values.

**The architecture of TransCell for drug sensitivity prediction**

In drug sensitivity prediction, the CCLE data was rescaled in the range of 0 to 1 and the target data was rescaled in the range of −1 to 1 by min–max scaler. The TransCell is comprised of two-step pre-trained CCLEenc and P. For the architecture of P, we used Adam as an optimizer (learning rate = 0.0005) with MSE loss. The input layer merging the output of two-step pre-trained CCLEenc had 256 neurons, followed by two hidden layers with 192 and 96 neurons. The activation function was set as a hyperbolic tangent for each layer. Moreover, to address the overfitting problem, except for the output layer, the regularization penalties were applied to the output and bias for each layer. We added dropout 0.50, 0.30, and 0.20 for the input layer and two hidden layers, respectively. In addition, early stopping was implemented in the training process to stop model training once the loss of validation started to increase. The optimal hyperparameters in the TransCell were searched by KerasTuner with 100 max trails. Before computing the evaluation metrics and Spearman rank correlation, we performed inverse transform on the predicted values.

**The architecture of TransCell for protein prediction**

In protein prediction, the CCLE data was rescaled in the range of 0 to 1 and the target data was rescaled in the range of −2 to 2 by min–max scaler. The TransCell is composed of two-step pre-trained CCLEenc and P. For the architecture of P, we used Adam as an optimizer (learning rate = 0.0001) with MSE loss. The input layer merging the output of two-step pre-trained CCLEenc had 512 neurons, followed by two hidden layers with 224 and 96 neurons. The activation function was set as a hyperbolic tangent for each layer. Moreover, to mitigate the overfitting problem, except for the output layer, the regularization penalties were applied to the output and bias for each layer. The dropouts were 0.55, 0.25, and 0.4 for the input layer and two hidden layers, respectively. In addition, we used early stopping in the training process. The optimal hyperparameters in the TransCell were searched by KerasTuner with 100 max trails. Before computing the evaluation metrics and Spearman rank correlation, we performed an inverse transform on the predicted values.

**The architecture of TransCell for copy number variation prediction**

In copy number variation (CNV) prediction, the CCLE data was rescaled in the range of 0 to 1 and the target data was rescaled in the range of −1 to 1 by min–max scaler. The TransCell is made up of two-step pre-trained CCLEenc and P. For the architecture of P, we used Adam as an optimizer (learning rate = 0.001) with MSE loss. The input layer merging the output of two-step pre-trained CCLEenc had 256 neurons, followed by two hidden layers with 256 and 32 neurons. The activation function was set as a hyperbolic tangent for each layer. Moreover, to address the overfitting problem, except for the output layer, we added the regularization on the output and bias for each layer. The dropouts were 0.60, 0.35, and 0.55 for the input layer and two hidden layers, respectively. Besides, we used early stopping to terminate the training process once the validation loss showed an increased trend. The optimal hyperparameters in the TransCell were searched by KerasTuner with 100 max trails. Before computing the evaluation metrics and Spearman rank correlation, we performed an inverse transform on the predicted values.

**The architecture of TransCell for mutation prediction**

In mutation prediction, the CCLE data was rescaled in the range of 0 to 1 by the min–max scaler. The TransCell consists of two-step pre-trained CCLEenc and P. For the architecture of P, we used Adam as an optimizer (learning rate = 0.001) with binary cross-entropy loss. The input layer merging the output of two-step pre-trained CCLEenc had 416 neurons, followed by two hidden layers with 192 and 32 neurons. The activation function was set as Rectified Linear Unit (ReLU) except for the output layer using the Sigmoid function. Moreover, considering the overfitting problem, except for the output layer, we added the regularization on weights for each layer. The dropouts were 0.05, 0.30, and 0.05 for the input layer and two hidden layers, respectively. In addition, we used early stopping in the training process. The optimal hyperparameters in the TransCell were searched by KerasTuner with 100 max trails.

**Feature selection methods**

Going from microarray to next generation sequence has given rise to a wealth of feature selection and dimension reduction techniques [9]. Feature selection methods are generally divided into three categories: filter methods, wrapper methods, and embedded methods [10]. The filter methods such as mutual information, Pearson correlation criteria, and Chi-square test are independent of any following learning algorithms. Due to the reduced computational time, filter methods are effective for high dimensional datasets; however, the nature that they consider each feature separately might weaken model performance [11]. Wrapper methods rely on learning algorithms to find a subset of features whose interactions would be considered during searching. Compared to filter methods, wrapper methods have higher computational costs and risk of overfitting [12,13]. Embedded methods combine the advantages of both filter and wrapper methods. Selecting the feature subset is considered as a part of model construction, implemented in many learning algorithms such as AdaBoost [14], RF, and decision tree [15,16]. The most common type of embedded methods is regularization methods, including LASSO [17], ridge regression [18], EN [19], regularized logistic regression [20], and logistic regression relying on the EN penalty [21].

LASSO regression performs regularization. The optimization problem is equivalent to the parameter estimation that follows:

(S1)

where *n* means the number of samples, , , and is the coefficient for feature *j*. penalizes the coefficients of the regression variables and shrinks some of them to zero. In this way, the variables that still have a non-zero coefficient after the shrinking process are selected to be essential features. The EN adds an additional regularization term into LASSO loss function. The EN loss function is shown as below:

(S2)

where and , which is a constant that multiplies the penalty term and is the elastic net mixing parameters with deciding a combination of and penalty. For instance, if , the penalty is a regularization. If , the penalty would be regularization. In other words, the EN can be seen as a linear combination of the LASSO and ridge penalty. Therefore, EN has similar behavior, shrinking model weights, as LASSO allows essential groups of correlated features to be selected. RF also offers a feature selection indicator. It uses variance of out-of-bag errors from permutations to compute the importance of each feature during the training process. Moreover, PCA is a dimensionality reduction technique that projects the data into a lower dimensional space and retains most of the variation part in the data set. By doing so, the most important information from the original data could be represented by fewer components. Moreover, in binary mutation data, Logistic_LASSO and Logistic_EN are applied for feature selection keeping informative features out of the original data by removing irrelevant and redundant ones. Given a dataset *X* (), which has *n* samples and *p* features, let standing for the binary target of each sample. The conditional probability distribution of the class label *y*, given a feature vector *x*, is as below:

(S3)

where denotes an observation made of *p* features. The weight vector and intercept are the parameters of the logistic regression model. The log-likelihood function associated with the learning samples based on the Equation (S3) is defined as:

(S4)

A maximum likelihood estimation of the model parameters *w* and *c* would be obtained by minimizing the Equation (S4). Therefore, for Logistic_LASSO, which employs a *l1* regularization term on the regression parameters, the loss function of the optimization problem is shown as below:

(S5)

Moreover, to Logistic_EN, the logistic regression with a combination of and regularization terms, its loss function is given as follows:

(S6)

where controls the strength of and regularizations.

**Different feature set combination comparisons**

Considering meta data for the clinical dataset [The Cancer Genome Atlas (TCGA)] and cell line dataset (CCLE), we ran the TransCell to build all metabolite models under different feature set combinations (original features *vs.* original features + age + gender) in the first experiment. The first one was the original feature set which we used to train the TransCell (5000 genes); and the second one was the feature set including 5000 genes, age, and gender features. To the gender feature, we gave 1 to female and 0 to male. For the age feature, at first, we separated the age information into four groups (for example, 0–16, 16–45, 46–65, and >65). Each group was transformed by one-hot encoding. By doing so, the feature size would be increased to 5005 features. From the experimental results in Figure S4A–C, we could find that two different feature sets lead to similar performance for TransCell metabolite predictions. The Wilcoxon rank sum test *P* values were not significant for MSE, RMSE, and Spearman rank correlation.

Afterward, we took disease lineage, cell type information, into account when conducting the second experiment (original features *vs*. original features + cell type features). There were 50 cell types from the union set in TCGA and CCLE datasets. We neglected samples with unknown cell type in CCLE. Therefore, there were 1429 and 15,028 samples for CCLE and TCGA, respectively. The cell type information was encoded by one-hot encoding. We added additional 50 features in our original feature set. Therefore, in total, there were 5050 features. Compared to the original feature set which we used to train the TransCell (5000 genes), we found that adding cell type information would not change a lot toward the training performance for TransCell metabolite predictions as shown in Figure S5A–C. Meanwhile, the Wilcoxon rank sum test *P* values were not significant for MSE, RMSE, and Spearman rank correlation as well. Overall, age, gender, and cell type information were not informative in this case.

Compared to our original method to choose feature genes for TransCell, we further did feature gene selection by considering immune genes, stromal genes [22], housekeeping genes [23], and overexpressed genes [24]. We removed immune, stromal, and housekeeping genes from our original 5000 feature genes. Then, we added overexpressed genes of four common cancers including lung, skin, breast, and ovary cancers between CCLE and TCGA datasets. By doing so, there were 4861 feature genes. Based on these 4861 feature genes, we performed the drug sensitivity prediction (N = 20). In Figure S6A–C, the blue and orange boxplot showed original and another feature gene selection method considering immune, stromal, housekeeping, and overexpressed genes, respectively. Consequently, two feature gene selection methods showed similar results with the Wilcoxon rank sum test *P* values not significant for MSE, RMSE, and Spearman rank correlation.

**Further application of clinical response prediction based on the TransCell concept**

To evaluate the two-step transfer learning model on the clinical response prediction, we implemented a pre-two-stage transfer learning framework. We firstly learned an encoder from the CCLE dataset, then transferred encoder’s weights to the second autoencoder as its weight initialization. The second autoencoder was trained by the pan-cancer TCGA dataset. It is noted that the training features are 5000 genes, which are the same as TransCell. Afterward, we extracted the two-step pre-trained encoder to link to a prediction feedforward network P. Our goal here is to use this framework, which is similar to TransCell, to predict clinical cisplatin drug response based on the TCGA dataset. The cisplatin TCGA dataset is from [25]. Based on the response evaluation criteria in solid tumors (RECIST) standard [26], we considered the clinical responses as two types, namely responder (including complete response and partial response) and non-responder (including stable disease and progressive disease). Therefore, it is a binary classification problem. Moreover, after removing the records of those patients who responded inconsistently to one drug during the course of treatment, there were 279 samples for cisplatin drug response prediction.

Similar to the TransCell, this framework comprises a two-step pre-trained encoderand P. For the architecture of P, we used Adam as an optimizer (learning rate = 0.0005) with binary cross-entropy loss. The input layer merging the output of two-step pre-trained encoder had 352 neurons, followed by two hidden layers with 224 and 64 neurons. The activation function was set as ReLU except for the output layer using the sigmoid function. Meanwhile, we added dropout 0.30 and 0.40 for the first and second layer of P, respectively. In addition, early stopping was implemented in the training process to stop model training once the loss of validation started to increase. The optimal hyperparameters in this framework were searched by KerasTuner with 100 max trails. In Figure S7, the area under curves (AUCs) were computed based on the five-fold cross-validation. For the clinical cisplatin drug response prediction, the framework built under TransCell’s concept had the average of AUC 0.921. However, based on the same architecture, if we trained the model directly without pre-two-stage transfer learning, the average of AUC would be 0.615, suggesting the importance of the transferred parameters.

**Cancer cell line drug response prediction comparison between TransCell and DeepDR based on genomics of drug sensitivity in cancer project**

Deep learning model to predict drug response (DeepDR) based on mutation and gene expression profiles of cancer cell or a tumor was proposed by Chiu and his colleagues [27]. DeepDR model applied gene expression and mutation data to predict drug response (log-scale IC50, IC50: half maximal inhibitory concentration) for cancer cell lines (CCLE) based on Genomics of Drug Sensitivity in Cancer (GDSC) project [28]. Their model contains (i) a mutation pre-trained encoder trained by TCGA, (ii) a gene expression pre-trained encoder trained by TCGA, and (iii) a drug response predictor network integrating the first two subnetworks. We referred to [24] for setting DeepDR architecture. To TransCell, it consists of a two-step pre-trained CCLE encoder and a prediction network P (please refer to Methods). TransCell was deployed to do the same task as DeepDR did in [24]. Therefore, we made drug response predictions for 265 drugs from the GDSC project by TransCell and DeepDR, respectively. Figure S10 suggests that TransCell has better performance. The RMSE of TransCell is significantly lower than DeepDR (*P* = 5.2*10-6, Wilcoxon rank sum test). We inferred the difference of performance is due to the consideration of the features sharing the same distribution between TCGA and CCLE which could avoid the negative transfer. Moreover, since TransCell makes the prediction based solely on gene expression, it mitigates the quantity of uncertainties caused by transferring the information coming from different types of data as well compared with DeepDR.

**Multi-task learning based on TransCell’s architecture for metabolite prediction**

Multi-task learning (MTL) aims at improving generalization by learning multiple tasks simultaneously. The MTL model transfers knowledge from one task to another wherever these tasks are related. It has been commonly utilized to address high dimensionality and small cohort size challenges. We further built a MTL model for metabolite predictions based on TransCell’s architecture as shown in Figure S8. However, sharing information with unrelated task might negatively impact performance. Compared to the MTL model, TransCell has lower RMSE with significant *P* value under the same 20 metabolite predictions as shown in Figure S9. In other words, without the prior knowledge of the relationship between metabolites, we observed negative transfer. Since the metabolites which users want to predict by TransCell web portal may not all be related, we did not consider the MTL model in our case.

**Finding patterns of model evaluation for each measurement type prediction**

At the preliminary stage, we used some random samples (N = 20) to help us do the model type selection. Based on the evaluation matrices, we then chose the model with the best performance and made scale-up predictions. Here, we ran all samples of proteins (N = 214) and metabolite (N = 225) on each type of model. EN had the best performance with the lowest RMSE with the five-fold cross-validation for all protein predictions. Moreover, TransCell outperformed others with the lowest RMSE for all metabolite predictions. However, for the rest of measurement type due to large sample size, instead of running all samples for different types of models, we tried to show some patterns with 20, 100, and 200 samples (N = 20, N = 100, N = 200). Along with the increase of sample size, TransCell consistently stood out with the lowest RMSE based on the five-fold cross-validation for gene effect score and drug sensitivity predictions. For the CNV prediction, LASSO had the best performance with the lowest RMSE. Furthermore, despite the expansion of sample size in mutation prediction, Logistic_LASSO kept holding the highest F1 score. By observing these patterns, we suggest using our originally proposed model to make scale-up prediction for each measurement type. The corresponding experimental results could refer to Table S2.

**References**

[1] Doan DM, Jeong DH, Ji S. Designing a feature selection technique for analyzing mixed data. 10th Annu Comput Commun Workshop Conf (CCWC) 2020:46–52.

[2] Zeng WZD, Glicksberg BS, Li Y, Chen B. Selecting precise reference normal tissue samples for cancer research using a deep  learning approach. BMC Med Genomics 2019;12:21.

[3] Zhuang F, Qi Z, Duan K, Xi D, Zhu Y, Zhu H, et al. A comprehensive survey on transfer learning. Proc IEEE 2021;109:43–76.

[4] Pan SJ, Yang Q. A survey on transfer learning. IEEE Trans Knowl Data Eng 2010;22:1345–59.

[5] Alshalali T, Josyula D. Fine-tuning of pre-trained deep learning models with extreme learning machine. Int Conf Comput Sci Comput Intell (CSCI) 2018:469–73.

[6] Hinton GE, Salakhutdinov RR. Reducing the dimensionality of data with neural networks. Science 2006;313:504–7.

[7] Chai T, Draxler RR. Root mean square error (RMSE) or mean absolute error (MAE)? — arguments against avoiding RMSE in the literature. Geosci Model Dev 2014;7:1247–50.

[8] Alexander DLJ, Tropsha A, Winkler DA. Beware of R2: simple, unambiguous assessment of the prediction accuracy of QSAR and QSPR models. J Chem Inf Model 2015;55:1316–22.

[9] Saeys Y, Inza I, Larranaga P. A review of feature selection techniques in bioinformatics. Bioinformatics 2007;23:2507–17.

[10] Khaire UM, Dhanalakshmi R. Stability of feature selection algorithm: a review. J King Saud University — Comput Inf Sci 2019;34:1060–73.

[11] Beniwal S, Arora JK. Classification and feature selection techniques in data mining. Int J Eng Res Technol 2012;1:1–6.

[12] Visalakshi S, Radha V. A literature review of feature selection techniques and applications: Review of feature selection in data mining. IEEE Int Conf Comput Intell Comput Res 2014:1–6.

[13] Bai L, Wang Z, Shao YH, Deng NY. A novel feature selection method for twin support vector machine. Knowl Based Syst 2014;59:1–8.

[14] Wang R. AdaBoost for feature selection, classification and its relation with SVM, a review. Phys Procedia 2012;25:800–7.

[15] Pirgazi J, Alimoradi M, Esmaeili Abharian T, Olyaee MH. An efficient hybrid filter-wrapper metaheuristic-based gene selection method for high dimensional datasets. Sci Rep 2019;9:18580.

[16] Ram M, Najafi A, Shakeri MT. Classification and biomarker genes selection for cancer gene expression data using random forest. Iran J Pathol 2017;12:339–47.

[17] Tibshirani R. Regression shrinkage and selection via the lasso. J R Stat Soc Series B Methodol 1996;58:267–88.

[18] McDonald GC. Ridge regression. WIREs Comput Stat 2009;1:93–100.

[19] Zou H, Hastie T. Regularization and variable selection via the elastic net. J R Stat Soc Series B Stat Methodol 2005;67:301–20.

[20] Cheng Q, Varshney PK, Arora MK. Logistic regression for feature selection and soft classification of remote sensing data. IEEE Geosci Remote Sens Lett 2006;3:491–4.

[21] Zakharov R, Dupont P. Ensemble logistic regression for feature selection. IAPR Int Conf Pattern Recognit Bioinform 2011:133–44.

[22] Yoshihara K, Shahmoradgoli M, Martínez E, Vegesna R, Kim H, Torres-Garcia W, et al. Inferring tumour purity and stromal and immune cell admixture from expression data. Nat Commun 2013;4:2612.

[23] Hounkpe BW, Chenou F, de Lima F, De Paula EV. HRT Atlas v1.0 database: redefining human and mouse housekeeping genes and candidate reference transcripts by mining massive RNA-seq datasets. Nucleic Acids Res 2021;49:D947–55.

[24] Axelsen JB, Lotem J, Sachs L, Domany E. Genes overexpressed in different human solid cancers exhibit different tissue-specific expression profiles. Proc Natl Acad Sci U S A 2007;104:13122–7.

[25] Ding Z, Zu S, Gu J. Evaluating the molecule-based prediction of clinical drug responses in cancer. Bioinformatics 2016;32:2891–5.

[26] Eisenhauer EA, Therasse P, Bogaerts J, Schwartz LH, Sargent D, Ford R, et al. New response evaluation criteria in solid tumours: revised RECIST guideline (version 1.1). Eur J Cancer 2009;45:228–47.

[27] Chiu YC, Chen HIH, Zhang T, Zhang S, Gorthi A, Wang LJ, et al. Predicting drug response of tumors from integrated genomic profiles by deep neural networks. BMC Med Genomics 2019;12:18.

[28] Yang W, Soares J, Greninger P, Edelman EJ, Lightfoot H, Forbes S, et al. Genomics of Drug Sensitivity in Cancer (GDSC): a resource for therapeutic biomarker discovery in cancer cells. Nucleic Acids Res 2013;41:D955–61.
