# Supplementary figures and images for: TransCell: In Silico Characterization of Genomic Landscape and Cellular Responses by Deep Transfer Learning
Source: Genomics Proteomics Bioinformatics. 2024 Sep 6;22(2):qzad008. doi: 10.1093/gpbjnl/qzad008 (PMC11378636; doi:10.1093/gpbjnl/qzad008)

ROC

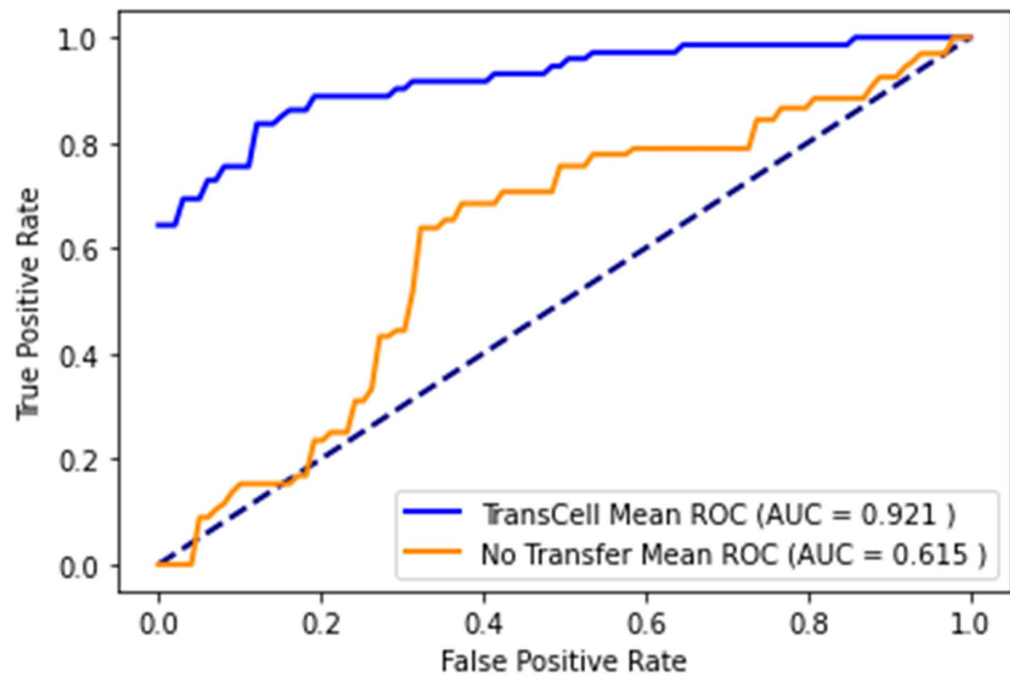

Supplement: qzad008_Supplementary_Data [file qzad008_supplementary_data.zip › FigS7.pdf]

# TransCell:

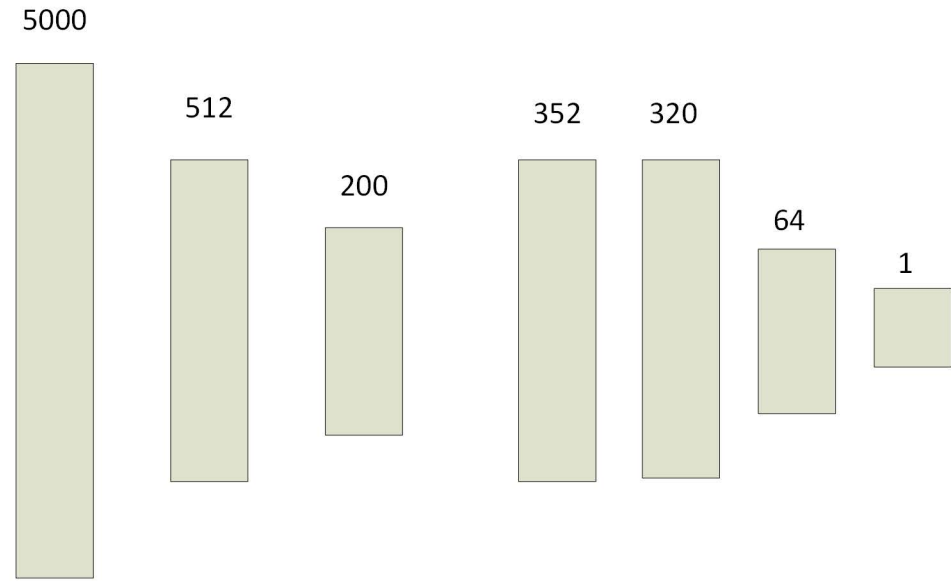

# Multi-task:

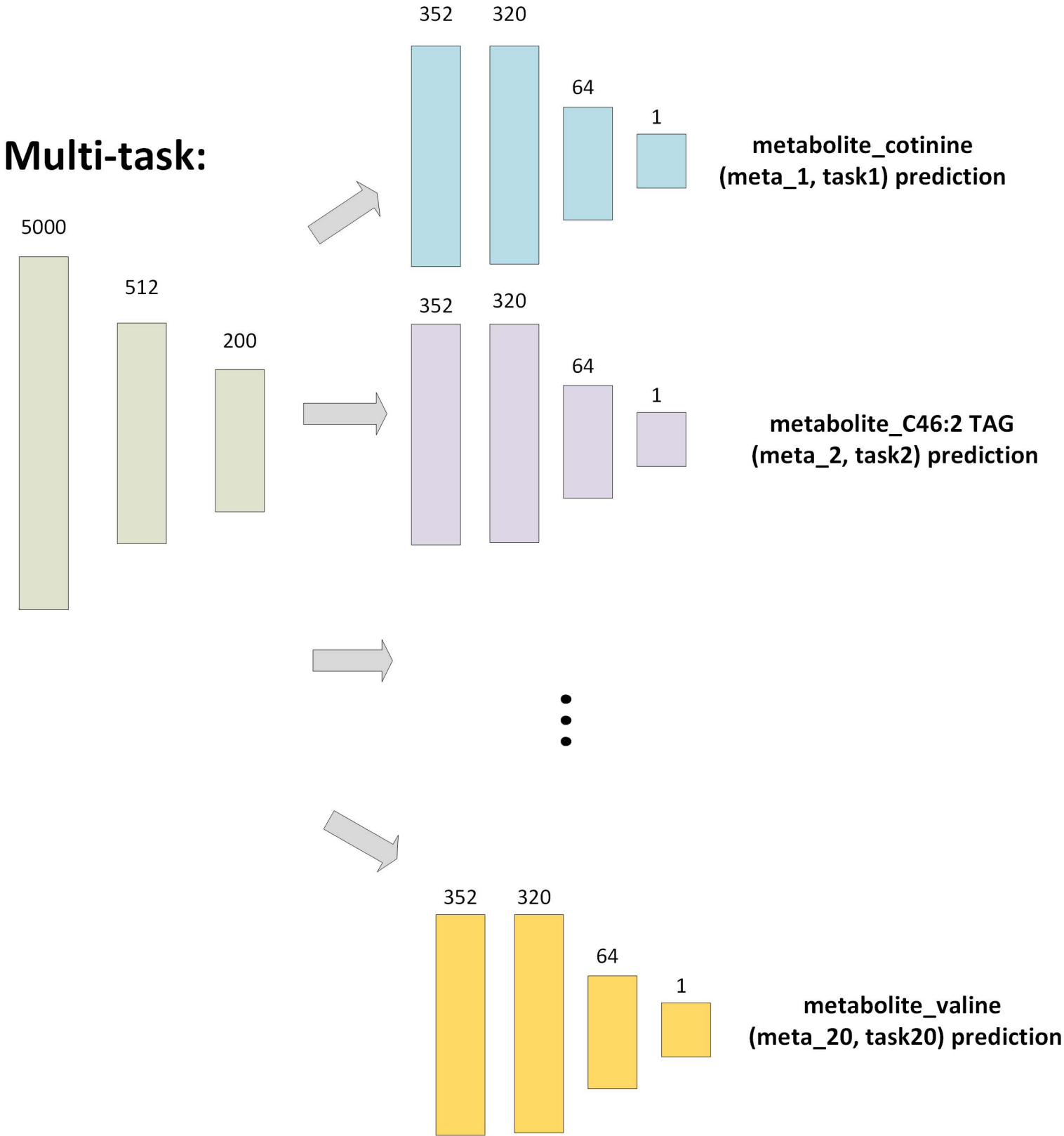

Supplement: qzad008_Supplementary_Data [file qzad008_supplementary_data.zip › FigS8.pdf]

Model type comparison (N = 20)

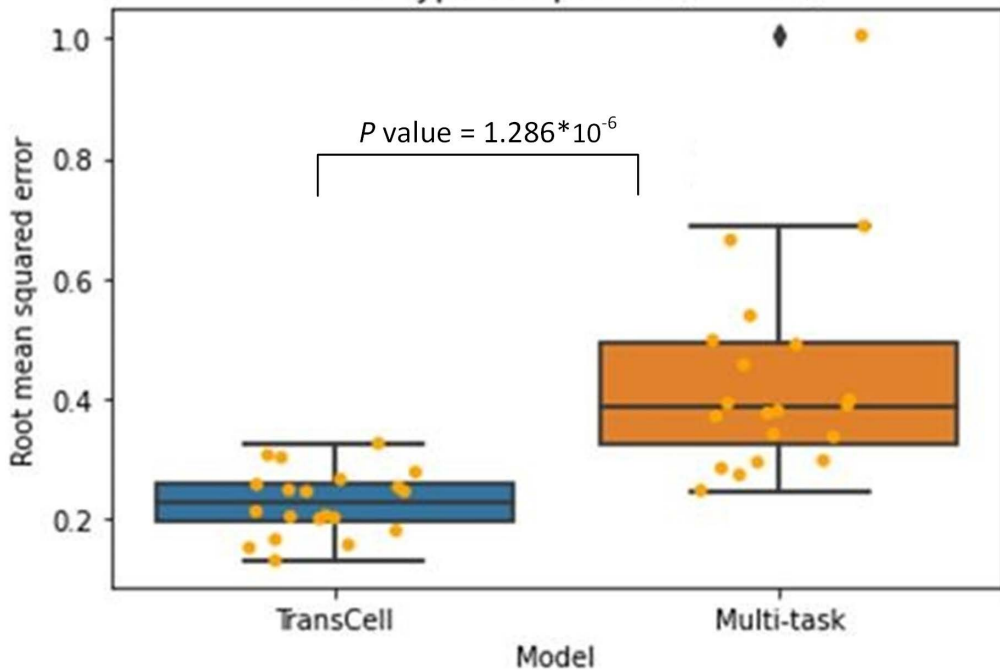

Supplement: qzad008_Supplementary_Data [file qzad008_supplementary_data.zip › FigS9.pdf]

Model comparison (GDSC drug prediction)

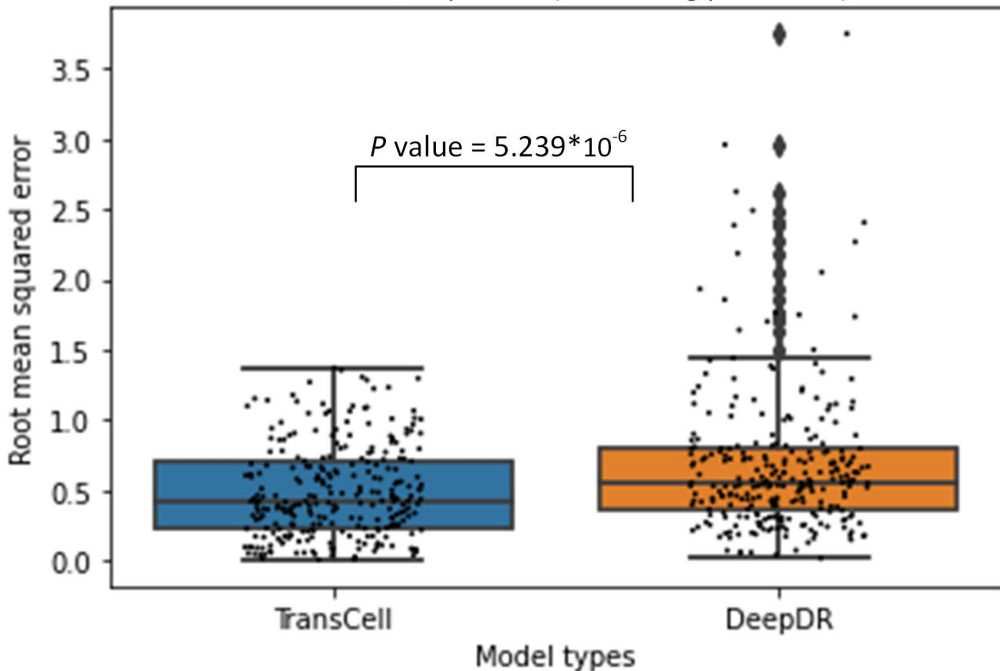

Supplement: qzad008_Supplementary_Data [file qzad008_supplementary_data.zip › FigS10.pdf]

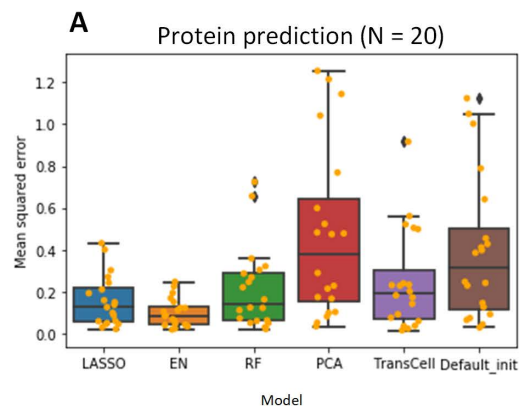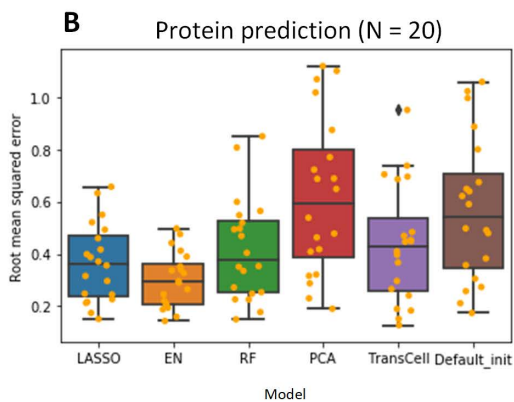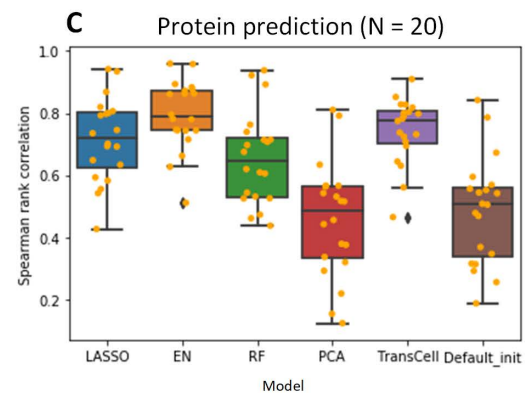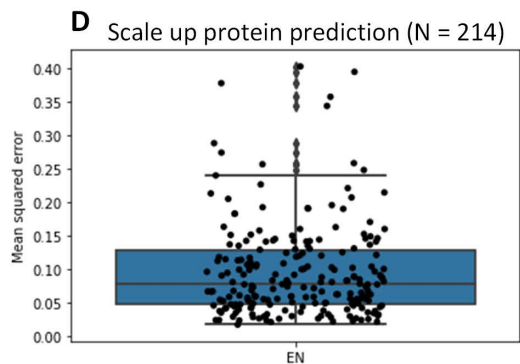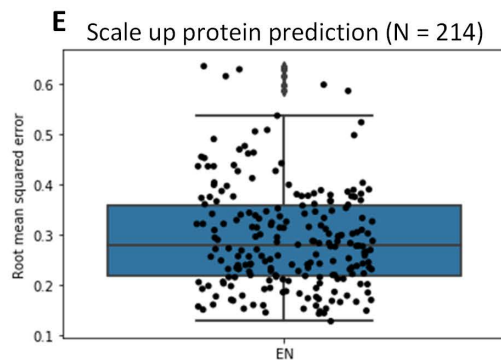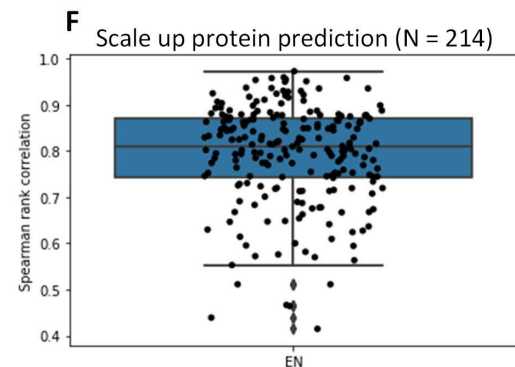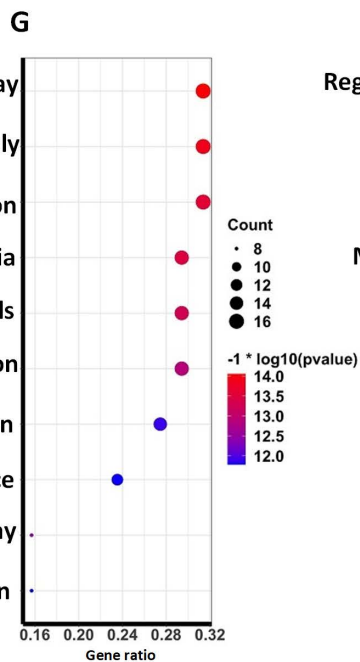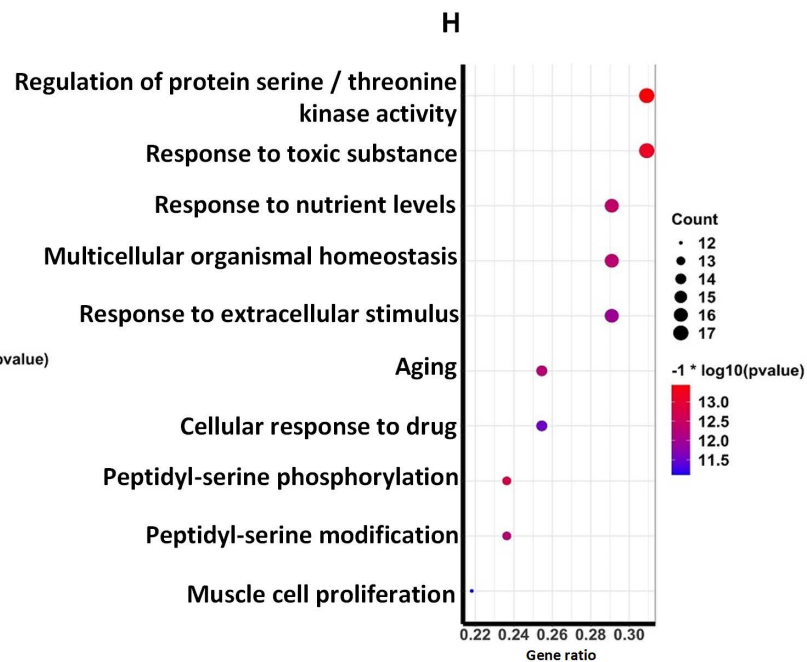

Supplement: qzad008_Supplementary_Data [file qzad008_supplementary_data.zip › FigS1.pdf]

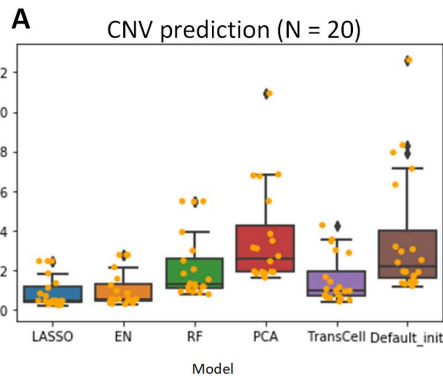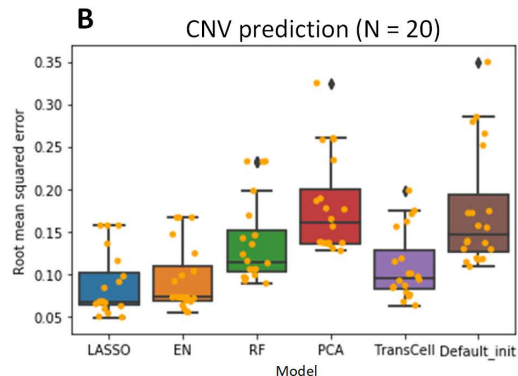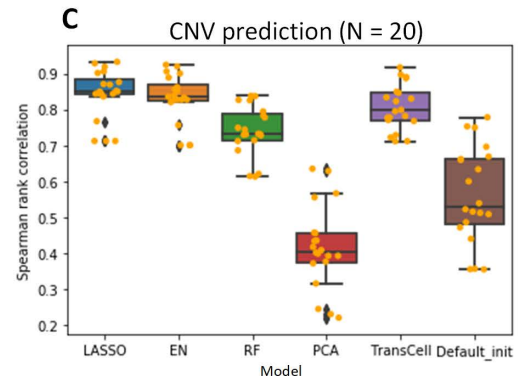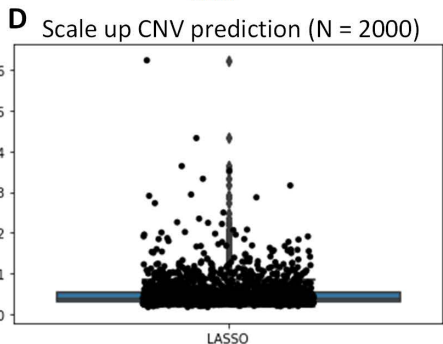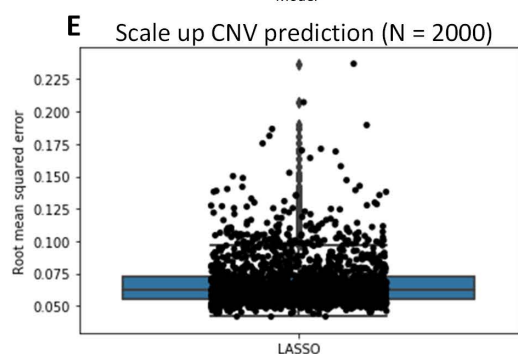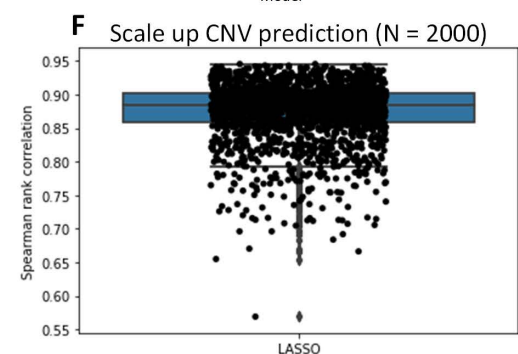

**G**

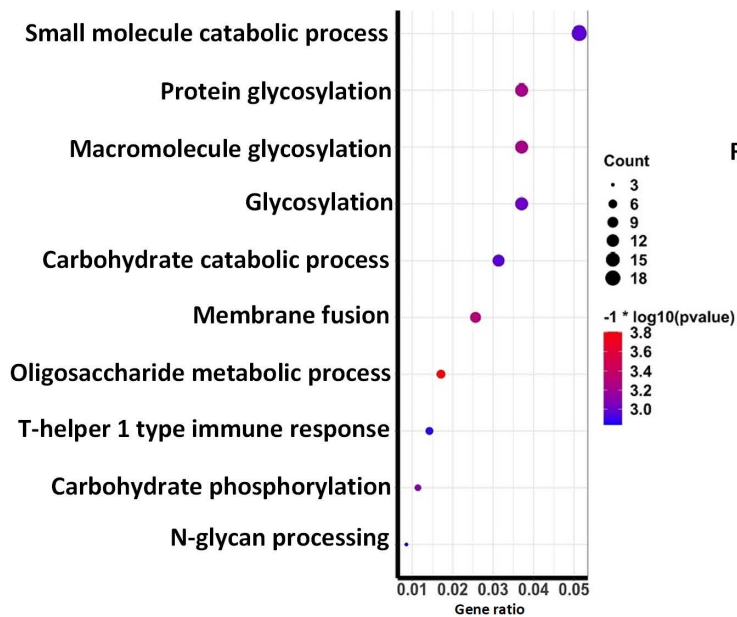

**H**

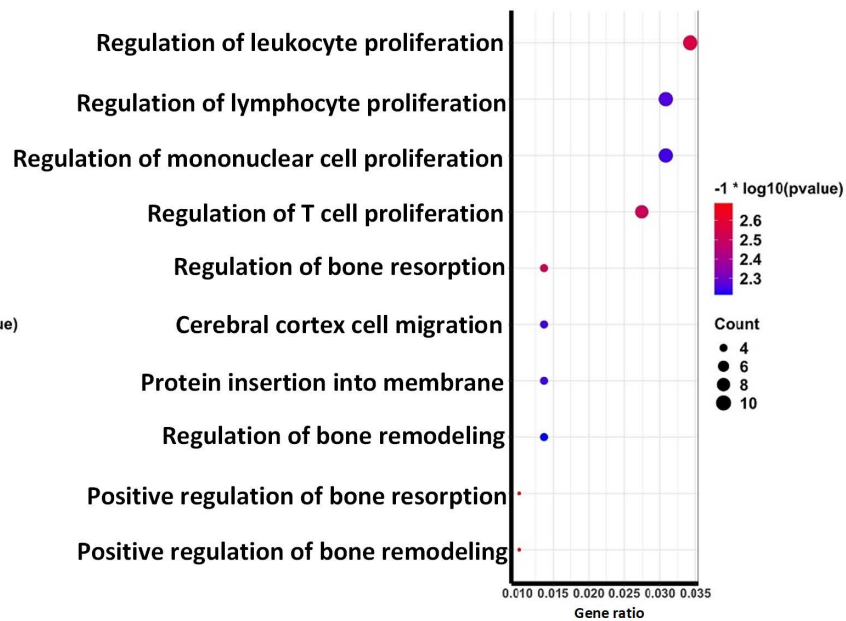

Supplement: qzad008_Supplementary_Data [file qzad008_supplementary_data.zip › FigS2.pdf]

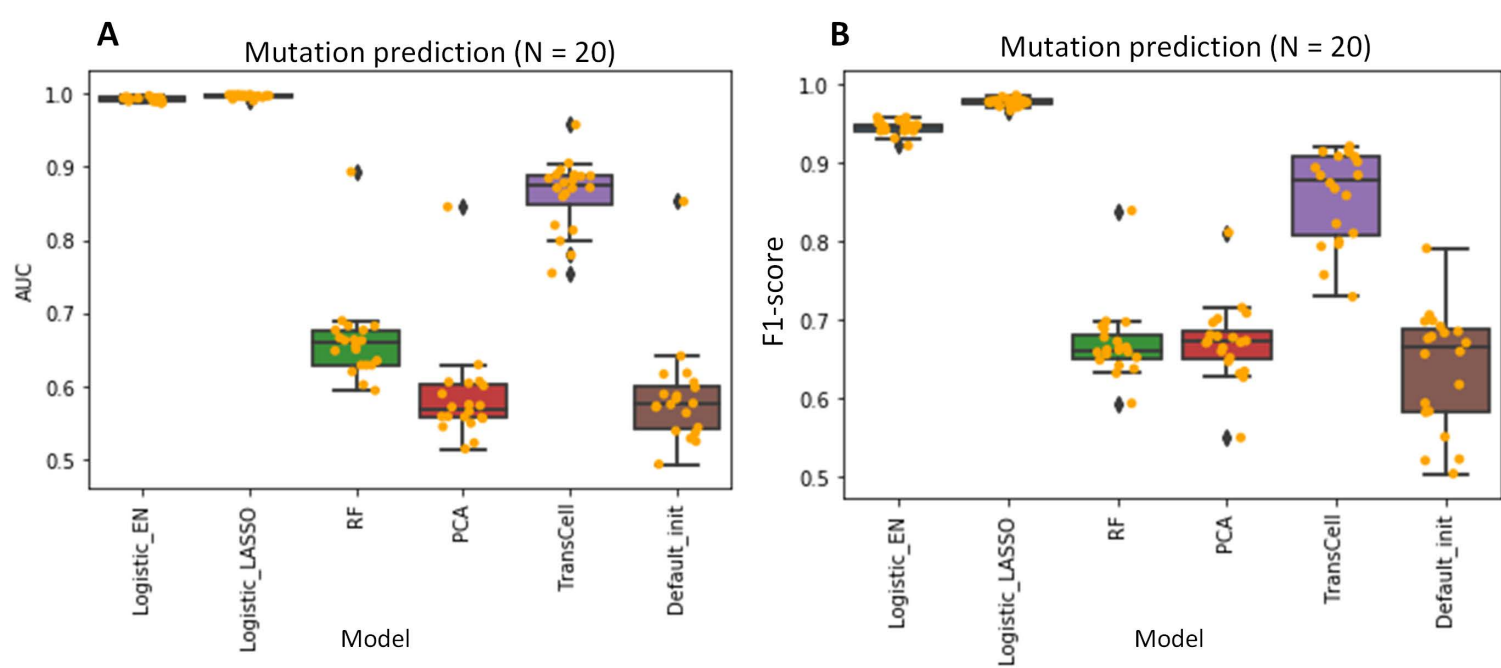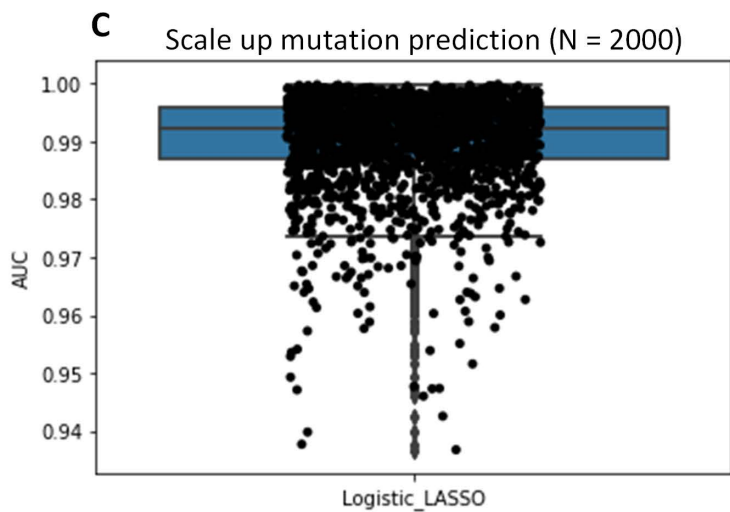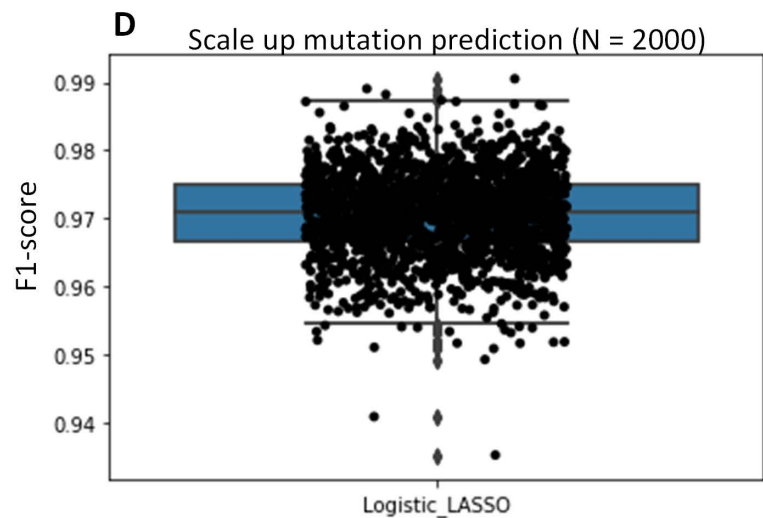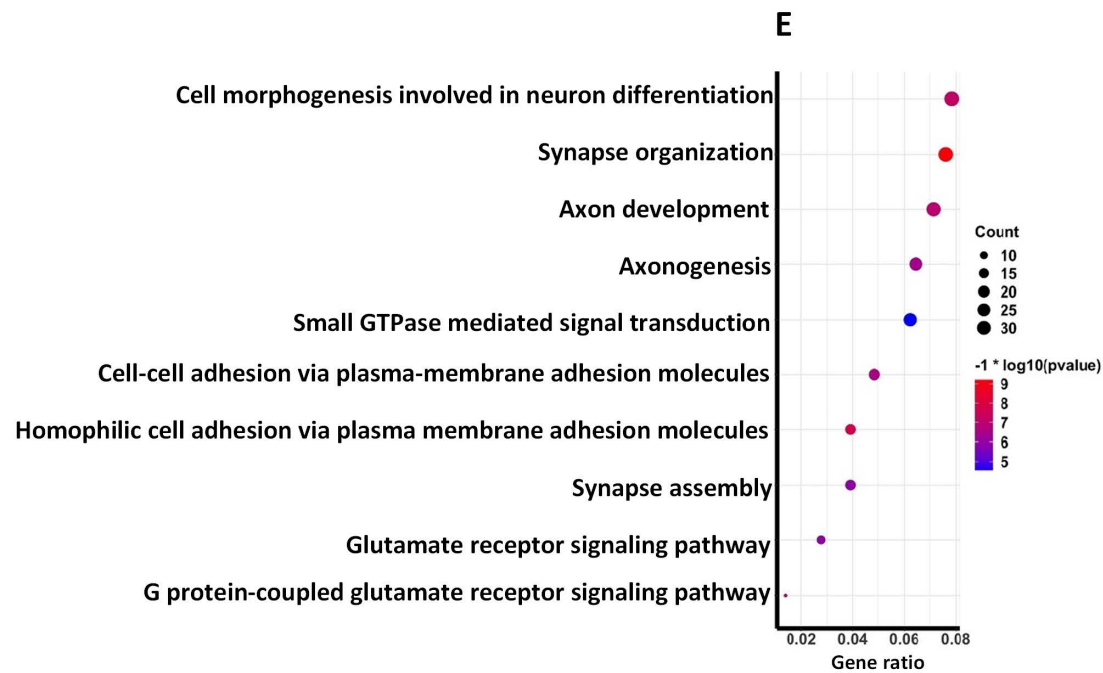

Supplement: qzad008_Supplementary_Data [file qzad008_supplementary_data.zip › FigS3.pdf]
